# Scalable quantification of dynamic subcellular spatial organization in single cells across tissues

**DOI:** 10.21203/rs.3.rs-9916651/v1

**Published:** 2026-07-02

**Authors:** Soumen Bera, Shalmali Pendse, Marcus Harrell, Jessica Nunes, Dirk Loeffler

**Affiliations:** 1 Department of Hematology, St. Jude Children’s Research Hospital, Memphis, TN 38105, USA; 2 Comprehensive Cancer Center, St Jude Children’s Research Hospital, Memphis, TN, USA.; 3 Department of Pathology and Laboratory Medicine, The University of Tennessee, Memphis, TN 38163, USA

## Abstract

The polarized organization of cellular constituents is vital for cell migration, fate decisions, and tissue organization and often altered in disease. However, its quantification remains challenging because cells vary in size, shape, marker expression, and change over time. As existing approaches lack throughput, discard spatial information, and cannot measure polarity dynamics, we introduce CellPolariS, a novel image analysis framework to quantify polarity in diverse cell types from different tissues and in living cells. Unlike other methods, CellPolariS uses spatial subcellular organization to quantify the number, direction, magnitude, and concentration of cellular structures. We identify that differences in cell morphology and marker expression between cells are critical confounding factors that impair polarity quantifications. Through extensive validation and quantification of diverse cell types, including T-cells, natural killer cells, zygotes, neurons, yeast, and bacteria, we demonstrate that CellPolariS corrects for these effects and provides precise measurements of the polarization of Tubulin, Actin, and other structures. In addition, we analyze hundreds of thousands of primary stem and progenitor cells, map how Tubulin, CDC42, and Actin polarity change throughout hematopoietic differentiation, and discover that polarity increases during erythroid differentiation. Using long-term imaging of living cells, we further show that polarized subcellular structures are highly dynamic and can quickly change from polar to non-polar states, suggesting that cell polarity is more dynamic than previously appreciated.

## Introduction

The polarized distribution of organelles, proteins, and RNAs is essential for cell and tissue function and regulates migration, signaling, and fate decisions, and is therefore of high relevance for understanding human health^1–3^. Polarity is often altered in diseases affecting epithelial^4^, but recent findings suggest that also hematopoietic stem and progenitors, and other cell types of the immune system use polarity^1^. However, as studies in invertebrates have shown, cell polarity is highly context-dependent, and the molecular regulation of polarity in many cell types remains poorly studied and entirely unknown for entire tissues^5^. Despite the biological importance of polarity, reliable tools to measure polarity in a standardized, easy-to-use way are lacking, preventing much-needed studies investigating how polarity affects disease progression, clonal evolution, tissue regeneration, and aging in single cells and entire tissues. However, these studies are currently not feasible as slow and error-prone manual inspection of a few select cell images is still the state of the art.^5^

Although measuring polarity may seem straightforward, its accurate and sensitive quantification at scale remains a major challenge in the field because (1) cells vary in size, shape, intensity, and marker distribution, (2) images vary in magnification, resolution, and bit-depth, and (3) true apolar and polar cells are lacking for comparison. Any reliable approach must thus consider and correct multiple biological and technical confounding factors, an unresolved challenge.

Most attempts thus far, including DeltaCentroid^6^, Polarity-JaM^7^, PolNet^8^, and CellDetail^9^, use pixel intensity averaging to reduce cellular marker distribution to a single simple ‘Polarity Score’, an approach that discards spatial information and limits sensitivity and accuracy. While alternative strategies that preserve spatial information, such as POlarity MEasurement (POME)^11^ have been developed, POME is complicated to use, works only in cells with regular hexagonal morphology, and limited in throughput as it is semi-automated. A reliable tool to quantify polarity robustly, reproducibly, and at scale is lacking, and a new standardized approach that works with any cell type, image format, and microscope is needed to understand how normal cells reversibly adapt to a plethora of context-dependent stresses and why these adaptations fail during chronic inflammation, aging, and diseases in different tissues.

We developed CellPolariS, the first fully automated and standardized algorithm capable of reliably quantifying polarity in any fixed or living cell. CellPolariS corrects for all confounding factors that impair the accuracy of previous methods^5^, including cell morphology, size, marker expression and distribution, magnification, and image bit depth. Rather than discarding spatial information, CellPolariS uses marker distribution to create intensity-weighted Polarity Vectors that encode marker direction, magnitude, and concentration, making this approach more sensitive. Unlike other methods, CellPolariS can (1) detect cells with one or more poles, (2) quantify relative pole positions, and (3) measure how polarity changes over time.

## Results

### Polarity Vector Quantification - a new, sensitive tool to quantify the number, magnitude, and orientation of cell poles in cell images

**Extended Data Fig. 1:**
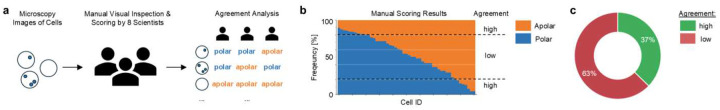
Manual scoring of cell polarity is slow and prone to human bias **a,** Experimental design. To determine the reproducibility of qualitative image analysis, eight scientists scored 50 cells as either polar or apolar by visual inspection. **b,** Summary of scoring results for each cell, showing the relative frequencies of polar and apolar annotations from different scientists. The presence of blue (polar) and orange (apolar) responses in the same cells indicates disagreement. **c**, Quantification of overall agreement and disagreement across all scored cells. Agreement was defined when at least 80% of annotators agreed. Manual scoring is not reproducible and prone to human bias.

Although never validated in detail, manual visual scoring of microscopy images remains the most widely used method to assess polarity. To quantify the reliability of this approach, we asked 8 scientists to score images of mouse hematopoietic stem and progenitor cells (HSPCs, Lineage-cKIT+Sca1+) stained for α-Tubulin and CDC42 as apolar or polar (Extended Data Fig.1). Although both α-Tubulin and CDC42 are well-established polarity markers^10,11^ scores varied widely (Extended Data Figs. 1b,c), indicating that manual polarity quantification is error-prone and not reproducible.

To overcome the limitations of existing tools, we systematically benchmarked the effects of cell size, morphology, magnification, marker intensity, and distribution on polarity scores. To avoid bias, we reasoned that simulating cells with defined size, shape, and fluorescence signals is a prerequisite for developing an accurate method to quantify polarity with high sensitivity in any cell type. This approach also allowed us to create completely apolar and polar reference cells to standardize results across different imaging platforms and to determine the reliability of polarity scores in cells with unusual shapes by simultaneously creating random permutations of multiple parameters.

**Fig. 1.**
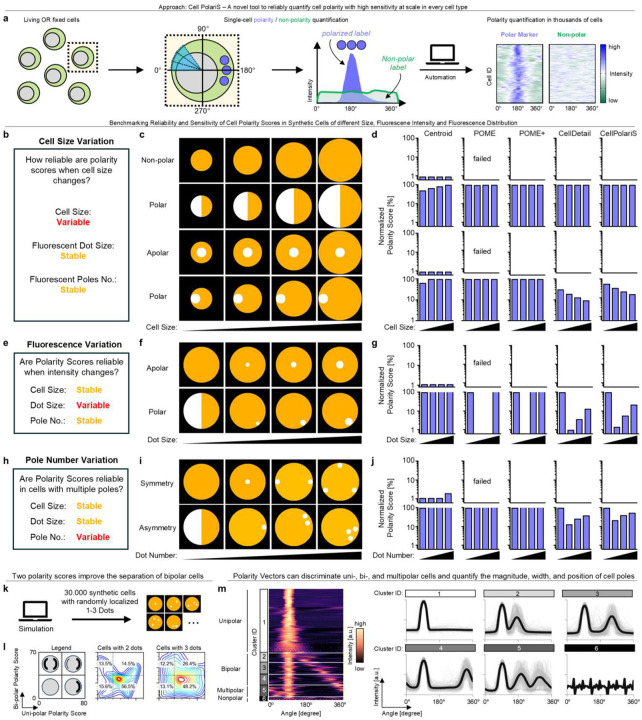
CellPolariS is more reliable, sensitive, and scalable than other polarity quantification approaches. **a**, Approach. CellPolariS can quantify cell polarity in any cell type by correcting several confounding factors, including cell morphology, cell size, marker expression, and distribution. To accomplish this, CellPolariS (1) transforms segmented cells into circular discs (morphology correction), (2) quantifies the mean intensity in adjustable 5-degree fractions in all directions (size correction), and (3) normalizes marker intensities on a per-cell level (intensity correction). Contrary to other approaches, which reduce the complex marker distribution into a simple polarity score, CellPolariS utilizes a ‘Polarity Vector’ that preserves the fluorescence intensities per angle, enabling more accurate and sensitive polarity analysis that can detect the number, magnitude, and orientation of cell poles. **b-j**, Comparison of CellPolariS polarity quantification, compared to other approaches, including Centroid, POME, POME+, and CellDetail. To compare different approaches, we normalized the polarity scores and created a CellPolariS polarity score to test how well approaches work in well-defined simulated scenarios of perfectly round cells. **b-d**, Quantification of cell polarity in cells with varying cell size, and constant fluorescence intensity and fluorescent poles. Reference cells with minimal and maximal possible polarity as displayed in the first two rows from the top. Despite identical marker distribution (white) in the cell with maximal polarity, the Centroid approach detects increases in polarity. POME failed to work in perfectly non-polar control and only worked after implementing improvements we call POME+. In polar cells with increasing cell size, only CellDetail and CellPolariS provide the expected reduction in polarity compared to Centroid, POME, and POME+. **e-g**, Quantification of cell polarity in simulated cells with increasing fluorescence intensities, and constant cell size and fluorescent poles. Only CellDetail and CellPolariS detected the expected increase in polarity when fluorescence intensity increased. **h-j**, Comparison of CellPolariS polarity score vs. other approaches, in simulated cells with one, two, or three cell poles when cell size and fluorescence intensity are stable. CellDetail and CellPolariS work as expected when multiple poles converge on one cell side. However, reducing cell polarity to a single number (“Polarity Score”) does not detect poles on opposite cell sides. Using simple Polarity Scores thus misses important information and can lead to misinterpretation. **k,** Experimental design. We simulated 30,000 perfectly round cells with randomly placed 1–3 fluorescent dots per cell to investigate how to reliably discriminate between cells with one, two, or multiple poles, as this cannot be accomplished using a single polarity score. **l,** Quantification of cell polarity of cells with 1, 2, or 3 fluorescent dots at random location, using two instead of one polarity score. To improve the separation of cells with different types of polarity and to reliably discriminate apolar cells from cells with two or more poles on opposite sides of the cells (see Figs. 1h–j, where a single polarity score fails), we combined CellPolariS’s uni-polar polarity score benchmarked in Figs. 1b–j, with a second polarity score (termed bi-polar polarity score) whose value increases when two or more poles are present in a cell. **m**, Although the use of two polarity scores combined improves polarity classification, even this approach cannot reliably distinguish different types of polarity in cells with two and three poles (compare Fig. 1l, middle and right), and does not preserve the magnitude, width, and relative orientation to multiple poles. To retrieve this information and to maximize cell polarity classification, CellPolariS therefore clusters Polarity Vectors (see heatmap), an approach that can reliably discriminate cells with one, two, or three poles as well as cells with poles of different sizes and relative orientation and position from the randomized and mixed pool of synthetic cells used in Fig. 1l. CellPolariS is therefore not only more sensitive than previous approaches, but it is also capable of detecting different types of cell polarity with unprecedented detail.

**Fig. 2.**
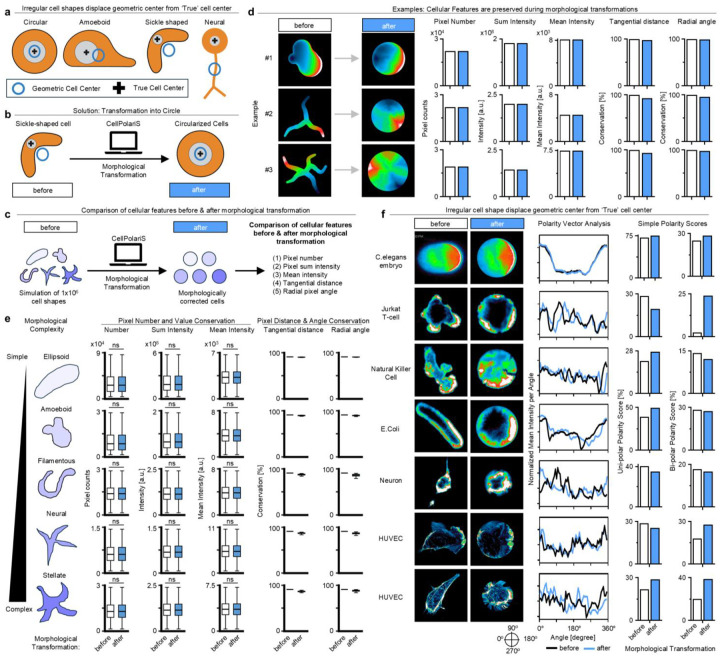
CellPolariS works in any cell type, irrespective of cell size and shape. **a**, Comparison between the geometric cell center and true cell center as reference points to measure cell polarity. Existing approaches use the geometric cell center, but irregular cell shapes can cause inaccuracies. In extreme cases, such as sickle cells or cells with long protrusions, the geometric center might be outside the boundary, leading to false positives. **b**, To overcome the issue, CellPolariS morphologically transforms cells into circular discs, where the geometric and true cell center overlaps. After transformation, the geometric cell center is now a reliable reference point to calculate polarity. **c**, To assess if circular transformation is reliable and can be used without altering the underlying data, we validated our approach by simulating over a million cells, ranging from cells with simple ellipsoids, amoeboid, and filamentous morphologies to more complex neural and stellate-like shapes. At the same time, we simulated different fluorescent levels and marker distribution to capture a broad spectrum of possible combinations of shapes and marker distribution. **d**, Representative images of simulated cells with amoeboid (#1), neural-like (#2), and stellate (#3) morphologies before and after circular transformation (left). Key cell features, including the total pixel number, pixel sum intensity, and pixel mean intensity, are completely unaltered during transformation, while at the same time, relative tangential and radial distance between adjacent pixels before and after transformation are preserved with high accuracy. **e,** Quantification of key cell features before and after morphological transformation in a million simulated cells with varying cell shapes, intensities, and marker distribution. Before and after comparison shows that total pixel numbers, pixel sum intensities, and pixel mean intensities are completely preserved irrespective of whether cells have a simple or complex cell morphology. Spatial distortions of relative distances (tangential and radial) of adjacent pixels are negligible in cells with ellipsoid and amoeboid shapes, and minor in cells with filamentous, neural, and stellate forms. Circular transformation is thus a reliable approach to correct cell shape-induced biases and errors during cell polarity quantifications. **f**, Cell morphology correction also improves the polarity quantification of real cells from various model organisms by removing cell shape bias. Examples of real cells with varying cell shapes are shown before and after transformation (left). Side-by-side comparison of different ways to quantify cell polarity shows that cell shapes can alter different types of polarity quantification, including our Polarity Vector approach and simple unipolar and bipolar scores. Correcting for irregular cell shapes is thus a prerequisite for accurate polarity quantification. STATISTICS: P values were determined by Welch’s two-tailed t-test in e; ns, not significant (P > 0.05); *P < 0.05; **P < 0.01; ***P < 0.001.

With this in mind, we developed CellPolariS, a new tool that overcomes these limitations. Contrary to older methods that reduce the complex cellular marker distribution to a single number and do not correct for cell morphology, CellPolariS (1) uses the spatial information by quantifying the fluorescence distribution in all direction in 360 degrees to preserve the angle (direction) of polarized proteins (Fig. 1a), (2) can distinguish between uni-, bi-polar and multipolar cells, (3) corrects for quantification artefacts caused by irregular morphologies, differences in cell size, and marker intensity, (4) uses imaging platform-independent reference cells to make results comparable, (5) provides an automated analysis pipeline, and (6) is the first tool that can measure polarity in single-cells over time.

**Extended Data Fig. 2:**
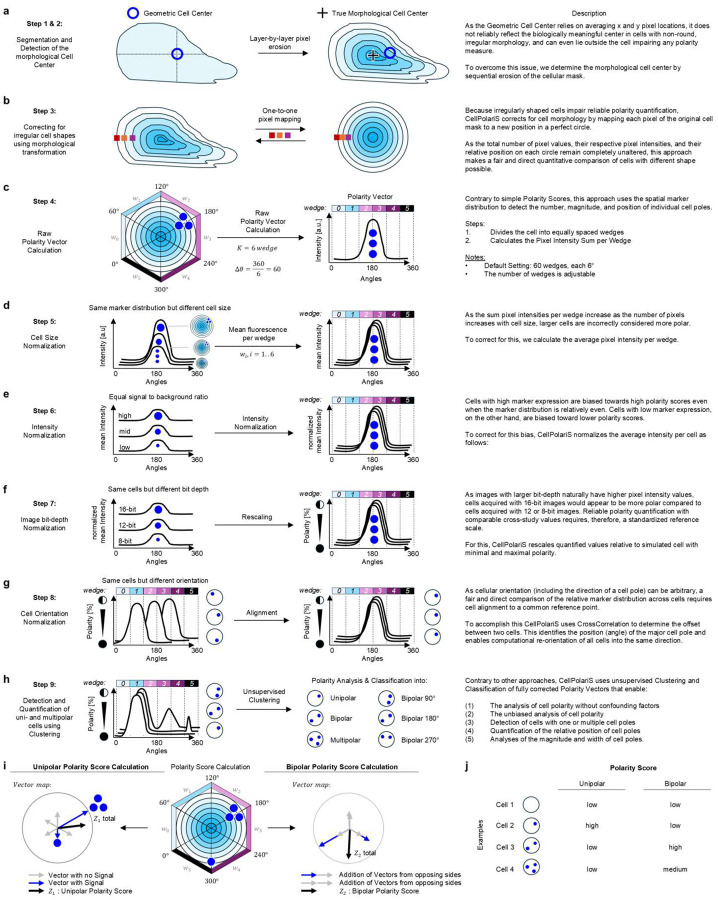
Step-by-step workflow for reliable cell polarity quantification using CellPolariS. **a,** Steps 1 & 2, After segmentation by a user-defined method, the geometric center is not a meaningful biological cell center for non-circular and irregular cell morphology. CellPolariS detects the morphological center by layer-by-layer erosion of the cell mask. **b,** Step 3, after defining the morphological center, CellPolariS corrects irregular cell shape into circular form by one-to-one pixel mapping from layer to layer, while maintaining parent-daughter relation. **c,** Step 4, CellPolariS then separates the circular cell into multiple equally spaced wedges corresponding to their position based on angle, where every wedge contains the sum intensity, called the polarity vector. **d,** Step 5, Per wedge total pixel intensity increases when cell size increases. To overcome this issue, CellPolariS normalizes the cell size by the sum of the mean intensity of every wedge. **e,** Step 6, Because cells with high marker expression are inherently biased toward higher polarity scores, CellPolariS corrects this effect by normalizing the mean intensity of each wedge using the within-cell intensity distribution. **f,** Step 7, Image bit-depth normalization. Images acquired at higher bit-depth have a larger intensity range and can therefore yield artificially higher raw polarity scores. CellPolariS corrects this effect by rescaling polarity values relative to simulated reference cells representing minimal and maximal polarity. **g,** Step 8, Because cell orientation, including direction of the cell poles, can vary arbitrarily, marker distributions were aligned to a common angular reference by cross-correlation with a Gaussian reference profile. The resulting angular offsets were used to rotate each cell before comparison. **h,** Step 9, Detection and quantification of uni- and multipolar cells by clustering.: After alignment, the primary pole of each cell was placed at a common reference angle (default, 90°). Secondary poles were then organized according to their clockwise angular positions, allowing bipolar cells to group by the location of the second pole and multipolar or weakly polarized cells to form separate clusters. This enabled quantification of the frequency of uni-, bi-, and multipolar cells, as well as the angular distribution of secondary poles in bipolar cells. CellPolariS also provides an average cell for each cluster for visualization. **I,** Detailed graphical representation of the Uni, and bipolar score calculation using polarity vector and circular geometry. The unipolar score is high when the signal is concentrated on one side of the cell and reports both the strength and direction of that pole. The bipolar score is high when the signal is concentrated in two opposite regions, whereas the unipolar score for the same pattern remains low because opposite sides cancel each other. Hemisphere polarity is calculated by comparing signal enrichment in the left and right halves of the cell, defined by a user-specified division axis.

**Extended Data Fig. 3.**
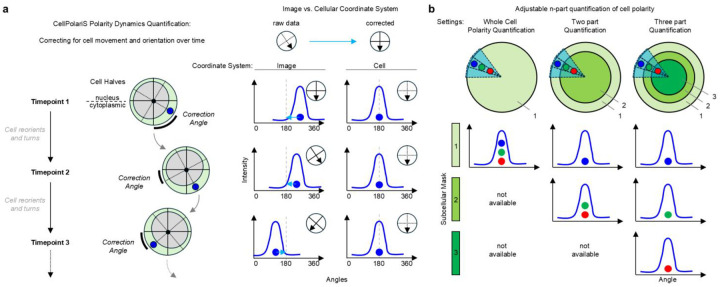
Correcting for changing cellular orientation over time is required for reliable Polarity Dynamics quantifications. **a,** Although CellPolariS corrects for many confounding factors found in images of fixed cells, CellPolariS can also be used to quantify changes in polarity dynamically over time. In these situations, cell movement between consecutive frames can change the cellular orientation, which might incorrectly appear as a moving cell pole. Cell Polarity Dynamics Quantifications, therefore, require additional correction. CellPolariS accomplishes this by using a reference channel to establish a cell-intrinsic coordinate system, whose rotation can be traced over time and compared to other polarity markers to discriminate changes in cell orientation from changes in marker localization within the cell over time. **b,** As different markers can localize and polarize to various cellular locations, CellPolariS allows quantification of cell polarity in different parts of the cells. This means, for instance, that the user can choose to split a cell into an outer and inner layer (of selectable size), to then compare, for instance, cortical polarity (outer layers) vs. cytoplasmic polarity (inner layers). Similar types of analysis can be done using 3, 4, or more layers to discriminate, for instance, membrane from cortical markers and cytoplasm. Calculating the cell polarity of specific layers can significantly boost the sensitivity of polarity measurements, which becomes increasingly more important with higher magnifications, when the cell membrane and/or cell cortex comprises only a small fraction of the overall cell signal.

To accomplish this, CellPolariS uses 9 steps, including segmentation, morphology-, cell size-, marker intensity-, bit depth-, and cell orientation-corrections, calculation of the polarized spatial distribution of fluorescence signals that preserve the direction (angle), height, width, number, and relative positions of fluorescence peaks and unsupervised clustering for polarity type classification(Extended Data Figs. 2a–h). Additional settings allow to compare the cell polarity in subcellular compartments (e.g., membrane vs. cytoplasm) and to correct for cell movement during live cell imaging experiments (Extended Data Fig. 3).

**Extended Data Fig. 4:**
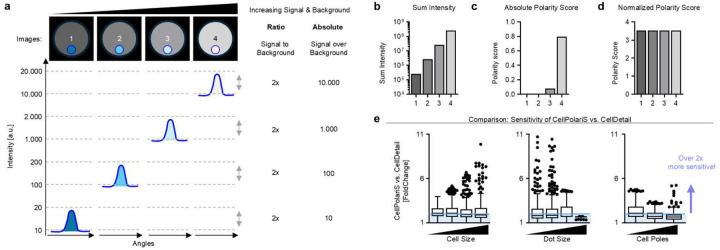
Intensity normalization - a prerequisite to reliably quantify polarity. **a-d**, Comparison of relative vs. absolute signal above technical and/or biological background and the effects of polarity quantification. **a,** Signal and background increase in tandem with increasing pixel dwell times, exposure, and/or excitation light. In this example, the signal-to-background ratio is constant, while the absolute difference between signal and background pixels increases. This can lead to over- and/or under-estimation of polarity in certain situations. Beyond image acquisition settings and microscopy hardware, this also means that without intensity normalization that corrects for these effects, polarity quantification is biased and overestimates the polarity of high-expressing cells while simultaneously underestimating the polarity of cells with low marker expression. **b**, Pixel sum intensity quantification of cells shown in **a**, demonstrating that increased marker expression in cells with identical fluorescence distribution and proportions leads to increased absolute polarity quantification (compare to **c**). **d,** Reanalysis of cells shown in **a**, after intensity-normalized polarity calculation, shows that the putative difference in polarity seen in **c**, is caused solely by marker expression differences. The cells depicted in **a**, therefore, do not differ in the relative cell polarity. **e**, Comparison of CellPolariS vs. CellDetail polarity quantification in different situations (cell size variation, Fluorescence size, and cell number variation) as shown in Figs. 1b–j. Quantification of 1000 simulated round intensity-normalized cells using CellPolariS and CellDetail polarity scores. Cells with varying size, fluorescent dot size, and number of dots scattered throughout the cells, show that CellPolariS polarity quantification is more sensitive than CellDetail in most situations.

To determine how CellPolariS compares to other approaches, we quantified how cell size, fluorescence intensity, localization, and distribution affect the Polarity Scores of other tools, including DeltaCentroid^6^, POME^12^, and CellDetail^9^ (Figs. 1b–d). To make the results of different approaches comparable, we used simulations of completely apolar and polar reference cells for standardization. We find that our score works more reliably and is more sensitive than DeltaCentroid, POME, POME+ (improved POME), and CellDetail in cells that differ in size (Figs. 1b–d), fluorescence distribution (Figs. 1e–g), number of fluorescent poles (Figs. 1h–j), and fluorescence intensity (Extended Data Figs. 4a–d). Our systematic comparison reveals that accurate polarity quantifications using DeltaCentroid, POME, and POME+ are impaired by multiple confounding factors. DeltaCentroid assigns larger polarity scores to larger cells (Figs. 1b–d); both DeltaCentroid and POME do not detect increases in polarity correctly when the fluorescence intensities (Figs. 1e–g) and signal distribution change (Figs. 1h–j). In comparison, CellPolariS and CellDetail quantify polarity correctly across different cell sizes, marker intensities, and distributions. However, direct comparison of CellDetail and CellPolariS shows that CellPolariS is more sensitive across all tested scenarios (Extended Data Fig. 4e).

However, although CellPolariS polarity score outperforms previous tools, we also discovered that reducing the cellular marker distribution to a single polarity score often leads to incorrect results. This occurs, for instance, when fluorescence signals on opposing sides of a cell cancel each other out in multipolar cells (Figs. 1i–j), as polarity scores cannot distinguish between non-polar cells and cells with multiple poles. We therefore simulated 30.000 synthetic cells with 1, 2, or 3 randomly placed fluorescent dots to test whether combining a second “Bi-polar Score” (BPS) with our primary “Uni-polar Score” (UPS) can improve the detection of multipolar cells (Figs. 1k,l). Indeed, using 2 scores instead of 1 can discriminate apolar cells (lower left quadrant) from cells with two or more poles (upper left quadrant) (Fig. 1l). It can also separate cells with a single pole (lower right quadrant) from cells with two poles of varying strength (right upper quadrant). However, even when using two scores, information about the number of poles, their magnitudes, widths, directions, and relative positions, which is critical when studying coordinated polarity within cell groups, tissues, and measuring dynamic polarity over time, is lost.

To enable these types of high-quality polarity analyses, we reasoned that using the information contained in the spatial marker distribution is critical and could be accomplished by using the fluorescence intensity vectors we calculate as the first step for our Polarity Score calculation. As these ‘polarity vectors’ preserve the spatial fluorescence intensity distribution in every direction around 360 degrees, they can also be used for unsupervised clustering, and remove human bias while enabling us to quantify the number, amplitude, and position of each fluorescence intensity pole to reliably distinguish between uni-polar, bi-polar, and multi-polar cells (Fig. 1a). To determine if polarity vectors improve sensitivity and quality of polarity quantifications, we reanalyzed the 30.000 simulated cells with randomly placed fluorescent dots (Figs. 1k,l) using unsupervised clustering of polarity vectors. As predicted, using the spatial information contained in cell images dramatically improves polarity analysis (Fig. 1m) and allows us to (1) separate cells with one, two, or three poles with high accuracy, and (2) detect cells with strong and weak poles at different relative locations, showing that polarity vectors outperform simple polarity scores dramatically.

#### Cell morphology correction is necessary for accurate quantification of cell polarity.

As cells are rarely perfectly round, we next sought to determine how cell shape affects polarity quantifications, as reliable and unbiased polarity quantifications must be independent of morphology. However, the impact of cell shapes on polarity quantifications has thus far been ignored, as most tools assume that cells are round. Consequently, the geometric cell center, which is commonly used as a reference point to assess the polar distribution of biomolecules, can be far from the actual cell center and even lie outside the cell boundaries, leading to unreliable results (Fig. 2a).

**Extended Data Fig. 5.**
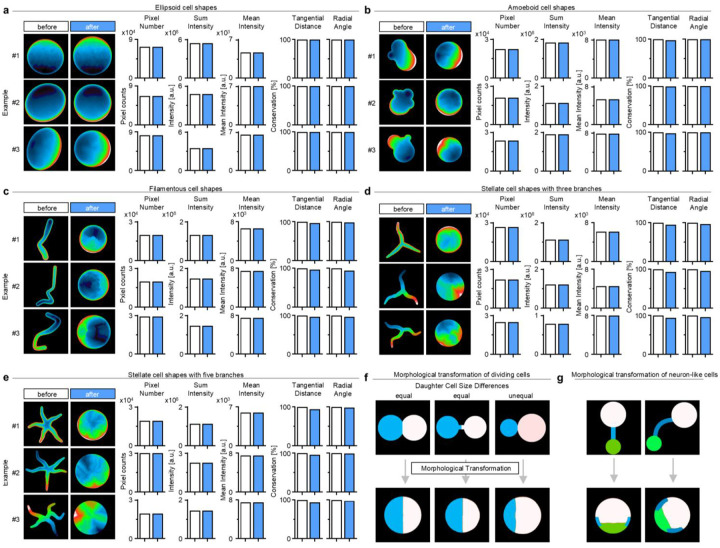
Circular transformation works reliably across a wide spectrum of complex cell morphologies, including neural shapes and cells during division. **a-e,** Representative images of cells with ellipsoid (a), amoeboid (b), filamentous (c), neural (d), and stellate (e) morphologies before and after circular transformation (left) and the effects on key cell features, including total pixel number, pixel sum intensity, mean pixel intensity, and relative tangential and radial distance between adjacent pixels. Pixel number, pixel sum intensity, and mean intensity are completely unaltered, while spatial distortion, measured based on the tangential and radial distances of adjacent pixels, is minor. Circular transformation thus does not meaningfully alter key cell features and is a reliable approach to correct errors introduced by irregular cell morphologies. **f,** Assessing the effects of circular transformation on cells captured early (left) and late (middle) during division, and in a cell that divides asymmetrically into a large and small daughter cell. CellPolariS circular transformation also works in dividing cells reliably. **g,** Analysis of how circular transformation affects cells with straight and bent neural-like morphology. CellPolariS circular transformation also works in cells with elongated morphologies, where two large cell parts are connected by a thin bridge.

To reliably correct for different cell shapes, CellPolariS (1) identifies the morphological cell center (contrary to geometric cell center) (Fig. 2a), and (2) transforms any irregularly shaped cell into a circular disk allowing a direct and fair comparison (Fig. 2b). To verify that the circular transformation does not alter the underlying data, we quantified pixel numbers, pixel intensities, pixel mean intensities, as well as the tangential and radial distance between cellular structures before and after transformation in simulated cells with ellipsoid, amoeboid, filamentous, neural and stellate shaped cells (Figs. 2c–e; Extended Data Figs. 5a–e). Our systematic and comprehensive quantification of over 1 million simulated cells with increasingly complex morphologies shows that the mathematical circular transformation works reliably without changing pixel numbers or intensity and has negligible effects on relative pixel positions even in cells with the most complex and irregular morphologies (Fig. 2e), including mitotic cells and cells with neural cell morphologies (Extended Data Figs. 5f,g).

To ensure that CellPolariS also works with real-world images of cells, we quantified irregularly shaped cells from the C. elegans embryo, Jurkat T-cells, natural killer cells, E. coli, neurons, and human umbilical vein endothelial cells (HUVEC) (Fig. 2f). As with simulated cells, polarity quantifications become increasingly unreliable in cells with complex shapes without cell morphology correction. Although the severity of these effects varies, even relatively minor changes in morphology, as seen in the examples for Jurkat T-cells, natural killer cells, and HUVEC, illustrate that both Uni-polar and Bi-polar Scores calculation can be greatly affected and must be corrected for reliable polarity quantification (Fig. 2f). Together, these results show that CellPolariS morphological correction is (1) a reliable and robust strategy to computationally correct for irregular cell morphologies, and (2) that correcting irregular cell shapes is a prerequisite for reliable polarity quantification.

### CellPolariS can detect changes in polarity induced by environmental stimuli and genetic perturbation with high sensitivity and throughput

**Fig. 3.**
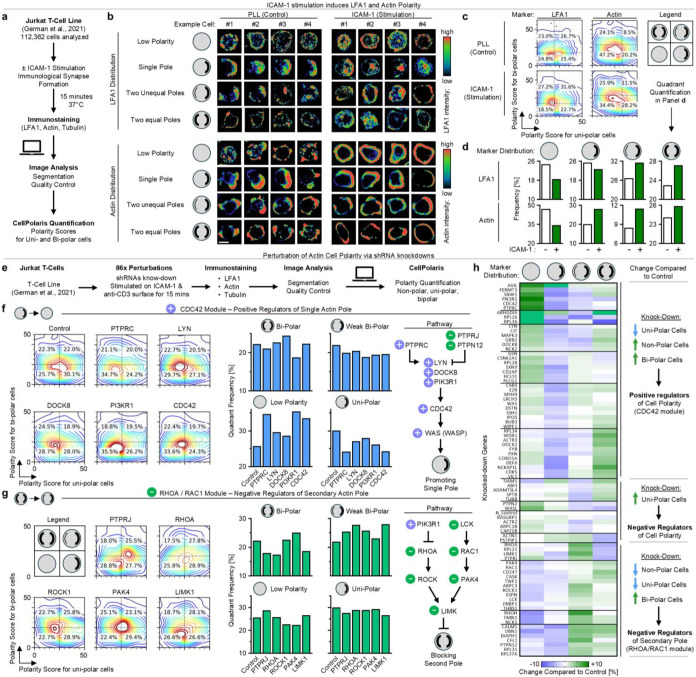
CellPolariS reliably identifies previously established and novel polarity regulators in sensitive high-throughput perturbation data. **a**, Experimental design. Jurkat T-cells were cultured on slides coated with either Poly-L-Lysine (PLL) or ICAM-1 to induce polarity, fixed, and stained for LFA-1, Actin, and Tubulin to quantify cell polarity using CellPolariS. **b**, Representative cells stained for LFA1 (top), and Actin (bottom) cultured on PLL (left) and ICAM-1 coating (right). Contrary to other approaches, which can only distinguish low vs. high polarity, CellPolariS enables the identification of cells with “Low Polarity”, “Single Pole”, “Two Unequal Poles”, and “Two equal Poles”, respectively. ICAM-1 induces redistribution and polarization of LFA1 and Actin. **c, d,** Quantification of LFA1 and Actin polarity using two Polarity Scores to identify cells with one (Unipolar Polarity Score) and two poles (Bipolar Polarity Score). As expected, ICAM-1 induces LFA1 and Actin polarity of Jurkat cells compared to PLL control. LFA1 increases the frequency of cells with two equal poles (top left quadrant) and two unequal poles (top right quadrant) while reducing cells with low polarity (lower left quadrant) and a single pole (lower right quadrant), and the frequency of cells with a single Actin pole increases (lower right quadrant). **e**, Experimental design. Jurkat T-cells were transfected with shRNAs to knock down 86 different regulators of immunological synapse formation, including many classical polarity regulators such as CDC42, RHOA, and RAC1. Stimulated on ICAM-1 & anti-CD3 surface for 15 mins after knock-down cells were fixed and stained for LFA-1, Actin, and Tubulin, to determine if CellPolariS detects the expected reduction of polarity after knock-down of established polarity regulators. **f-h**, Quantification of Actin polarity changes after knockdown of established polarity regulators. **f,** Knockdown of positive up- and downstream regulators of CDC42 reduces cells with uni-polar actin polarity (lower right quadrant). **g**, Knockdown of positive up- and downstream regulators of RHOA and RAC1 reduces the frequency of cells with equally strong Actin poles (upper left quadrant) and increases the number of cells with two Actin poles of different strength. **h**, Summary of changes in actin polarity after 86 knockdown experiments relative to no knockdown control cells. We detect at least three distinct clusters, including (1) Positive regulators of cell polarity genes are up- and downstream of CDC42, (2) Negative regulators of a secondary actin pole that positively regulate RHOA and RAC1 signaling, and (3) negative regulators of cell polarity.

To establish that CellPolariS works reliably, we analyzed Jurkat cells stained with Actin, LFA-1, and Tubulin^13^, which were stimulated with ICAM-1, a coating that induces adhesion, polarity, and immunological synapse (IS)-like organization^14^, and analyzed 86 shRNA-mediated knockdowns, including the polarity regulators CDC42, RAC1, and RHOA. Reanalysis of these data showed that ICAM-1 increases the polarity of Actin and LFA-1 as expected (Figs. 3a–d). We also find that knockdown of the pro-polarity factor CDC42 or its up- and downstream regulators, including PPTRC, DOCK8, and PIK3R1, reduces polarity (Fig. 3f). Also, knockdowns of either RAC1 or RHOA, which are often found on the opposite side of the cells, change polarity as expected (Figs. 3g–h). However, instead of reducing polarity, knockdown of these genes induces a secondary cell pole, something that would have been missed using a polarity score alone. Using clustering, we identify three major clusters that consist of up- and downstream regulators of the well-known polarity factors CDC42, RHO/RAC1, and genes that negatively regulate polarity (Fig. 3h). Together, this shows that CellPolariS faithfully captures the effects of well-known polarity regulators, demonstrating that CellPolariS works reliably with high sensitivity and throughput.

### Systematic analysis across the hematopoietic system reveals dynamic changes of CDC42 and Actin polarity during differentiation

**Fig. 4.**
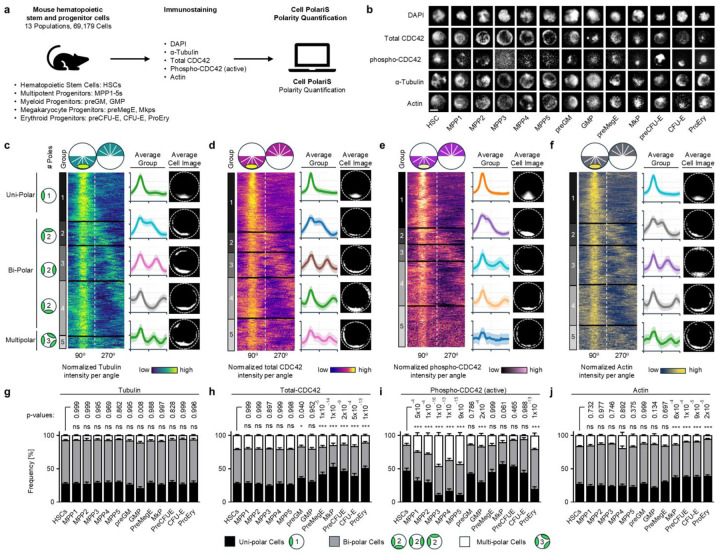
Hematopoietic stem cells and lineage-restricted progenitors are more polarized than multipotent progenitors. **a**, Experimental design. To compare how polarity changes during HSC differentiation, thirteen mouse hematopoietic stem and progenitor populations were freshly isolated, fixed, and stained for DAPI, Tubulin, total CDC42, phospho-CDC42, and Actin, and quantified with CellPolariS. **b**, Representative images of different HSPCs stained for DAPI, Tubulin, CDC42, phospho-CDC42, and Actin. Scale bar: 10 μm. **c-f,** Left: Hierarchical clustering of single-cell polarity vectors for Tubulin (c), total-CDC42 (d), phospho-CDC42 (e), and Actin (f) in all 13 HSPCs. Clustering reveals five major groups, representing cells with one (Group 1: unipolar), two (Group 2–4: bipolar), and more than two poles (Group 5: multipolar). Cells with two poles are further discriminated based on the relative orientation of the two poles, which can be at a 90°, 180°, or 270° angle for Groups 2–4, respectively. Middle: Average Polarity Vector for Groups 1–5 for Tubulin (c), CDC42-total (d), phospho-CDC42 (e), and Actin. Right: Calculated average image of all cells within each group. HSPC polarity is heterogeneous, and cells can have one, two, or multiple poles. **g-j,** Relative frequency of cells with one, two, and multiple poles across 13 analyzed HSPCs. Tubulin polarity is comparable across all tested 13 HPSCs, while the Megakaryocytic-Erythroid progenitors have more unipolar cells for CDC42-total, phosphor-CDC42, and Actin. Compared to HSCs, unipolar cells are less common for MPPs, while the frequency of multipolar cells increases (i). Cell Polarity changes throughout differentiation from HSCs to lineage-restricted progenitors. n = 8 independent replicates, with 8801, 10539, 4583, 4422, 10689, 8609, 9043 and 12489 analyzed HSCs, MPP1s, MPP2s, MPP3s, MPP4s, MPP5s, preGMs, GMPs, preMegEs, Mkps, preCFU-E, CFU-Es, and Pro-Erys, respectively. P values were determined by Tukey-corrected Two-way ANOVA in g–j; ns, not significant (P > 0.05); *P < 0.05; **P < 0.01; ***P < 0.001.

To investigate how polarity changes during HSC differentiation, we used CellPolariS to analyze polarity in 13 freshly isolated HSPC populations^15^, including hematopoietic stem cells (HSC), different multipotent progenitors (MPP1–5), pre-Granulocyte-Monocytes Progenitors (preGM), Granulocyte-Monocyte Progenitor (GMP), pre-Megakaryocyte-Erythrocyte progenitor (preMegE), Megakaryocyte Progenitor (MkP), pre-colony-forming unit-erythroid (preCFU-E), colony forming unit-erythroid (CFU-E), and pro-erythroid (pro-Ery) (Supplementary Fig. 1). We isolated, fixed, and analyzed 69,179 HSPCs stained with DAPI, anti-β-Tubulin, anti-CDC42(total), anti-phospho-CDC42(S71), and anti-β-Actin (Figs. 4a–b). While previous reports show that Tubulin and CDC42 can polarize in HSCs^11,16^, how this polarity compares with that of other hematopoietic progenitors has not been investigated.

**Extended Data Fig. 6.**
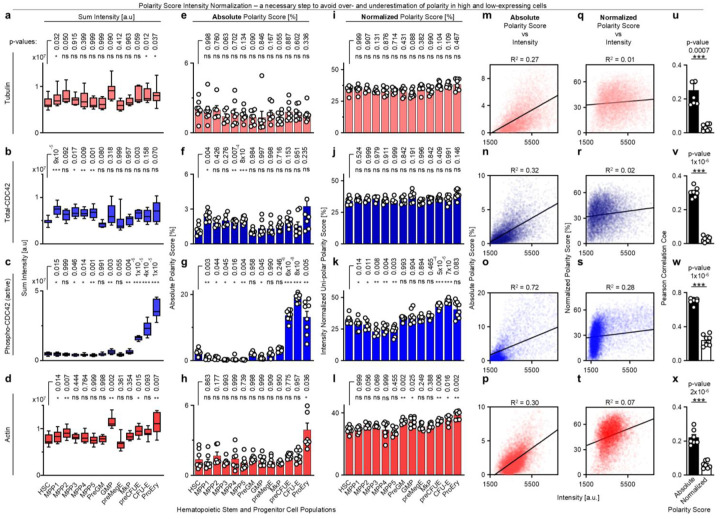
As cells with high expression levels are biased towards high polarity scores, intensity normalization is a prerequisite for reliable polarity quantification. **a-d,** Quantification of mean protein expression levels of Tubulin, Total-CDC42, phospho-CDC42, and Actin in 13 freshly isolated mouse HSPCs, including HSCs, MPP1–5s, preGM, GMPs, preMegE, Mkp, preCFU-E, CFU-E, and ProEry. While tubulin and total-CDC42 levels are comparable throughout the hematopoietic hierarchy, phospho-CDC42 and Actin protein levels increase in erythroid cells (preCFU-E, CFU-E, and ProEry). **e-h,** Quantification of absolute Polarity Scores for Tubulin, Total-CDC42, phospho-CDC42, and Actin in 13 freshly isolated mouse HSPCs, including HSCs, MPP1–5s, preGM, GMPs, preMegE, Mkp, preCFU-E, CFU-E, and ProEry. While tubulin polarity is comparable across all analyzed populations, the absolute polarity score is higher for cell populations with higher expression levels, including MPP1–5 for total-CDC42, preCFUE, CFU-E, and ProEry for phospho-CDC42, and ProEry for Actin, compared to HSCs. Mean ± SEM. **i-l,** Quantification of intensity-normalized polarity scores for Tubulin, Total-CDC42, phosphor-CDC42, and Actin in 13 freshly isolated mouse HSPCs, including HSCs, MPP1–5s, preGM, GMPs, preMegE, Mkp, preCFU-E, CFU-E, and ProEry. PreCFUE, CFU-E, and ProEry have higher Actin and phospho-CDC42 polarity than HSCs. Mean ± SEM. **m-p,** Absolute marker expression level and absolute polarity scores are strongly correlated for all tested markers, including Tubulin, total-CDC42, phospho-CDC42, and Actin. This shows that cells with high marker expression are biased towards higher polarity scores, while the polarity of low-expressing cells is underestimated. **q-t,** Contrary to m-p, intensity normalization of polarity scores does not correlate with marker expression level, while at the same time, cells with high and low (normalized) polarity are found. Marker intensity normalization is thus a prerequisite to quantifying cell polarity reliably. **u-x,** Quantification of Spearman Correlation between marker expression levels and absolute and intensity-normalized polarity scores. N = 8 independent experiments. P values were calculated using one-way ANOVA followed by Tukey’s multiple-comparisons test in a–l and Welch’s t-test for the panels u-y; ns, not significant (P > 0.05); *P < 0.05; **P < 0.01; ***P < 0.001.

**Extended Data Fig. 7.**
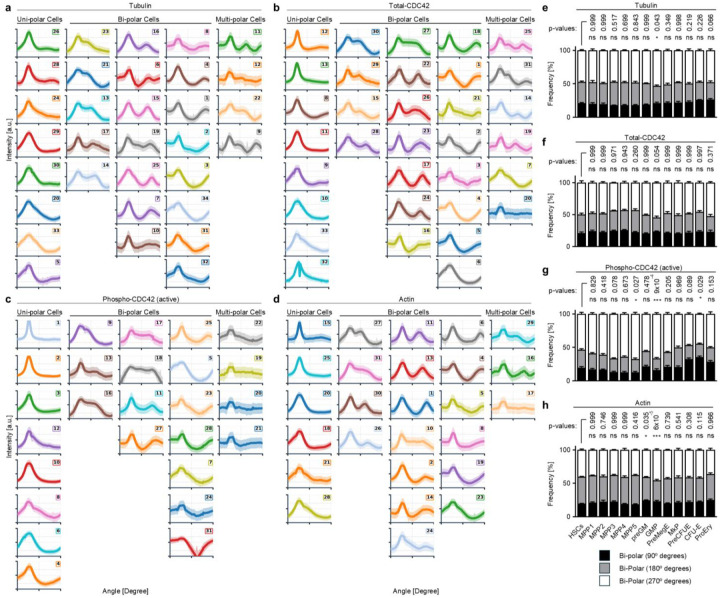
The majority of primary and secondary cell poles orient perpendicular to each other in hematopoietic stem and progenitor cells. **a-d,** Overview of all Tubulin, Total-CDC42, Phospho-CDC42, and Actin clusters used to classify uni-polar, bi-polar, and multi-polar cells in Figs. 4c–j. Mean normalized intensities per angle are shown as bold colored lines, shaded area indicates standard deviation. **e-h,** Relative frequencies of bipolar cells in which primary and secondary cell poles are oriented at 90, 180, and 270 degrees to each other. Overall, the relative orientation of primary and secondary cell poles is comparable between different hematopoietic cell types. However, all analyzed hematopoietic cells are enriched for primary and secondary poles with perpendicular orientations (90 ° and 270° angles), and only a small proportion of cells have the secondary pole on the opposite side of the cell (180-degree angle). P values were calculated using Two-way ANOVA followed by Tukey’s multiple-comparisons test. in e-h; ns, not significant (P > 0.05); *P < 0.05; **P < 0.01; ***P < 0.001.

As different cell populations vary substantially in cell size, morphology, and marker expression, correcting for confounding factors is critical for reliable polarity quantifications (Extended Data Fig. 6). We find that correcting for expression levels is particularly important, as high expressers are, irrespective of the marker, strongly biased towards high polarity scores, while the polarity of low expressers is underestimated (Extended Data Figs. 6c,g,m–p). After removing all confounding factors, Tubulin and CDC42-total polarity are comparable across all 13 HSPCs (Extended Data Figs. 6i–l), while phospho-CDC42 polarity (classically polarity-associated) is high in HSCs and erythroid progenitors and lower in MPPs1–5 (Extended Data Fig. 6k). As relying on Polarity Scores can be misleading (Figs. 1h–m, Fig. 2f), we analyzed these 13 populations by clustering Polarity Vectors. Using the magnitude and marker distribution of this approach, we find cells with one, two, or multiple poles, and bipolar cells with poles at 90°, 180°, or 270° angles (Figs. 4c–f), (Extended Data Figs. 7a–d). While the number of Tubulin and CDC42-total poles is comparable across all tested HSPCs, unipolar cells for phospho-CDC42 and Actin are more abundant in PreMegE, MkP, PreCFU-E, CFU-E, and Pro-Ery progenitors compared to HSCs (Figs. 4g–j). Together, this analysis shows (1) that polarity changes throughout differentiation from HSCs, MPPs, to late progenitors, and (2) suggests that during this process, HSPCs acquire two or more poles rather than losing polarity.

To further investigate why bipolar cells have poles at different angles, we reasoned that if the angles regulate cell function, specific bipolar angles should be enriched, while a random distribution would make a regulatory role unlikely. Our analysis shows that secondary poles are frequently positioned at roughly a 90° angle (90° and 270°) relative to the main pole, while relatively few cells have poles on the opposite side of the cell (grey bars; 180°), suggesting the relative position of poles might carry meaning (Extended Data Figs. 6e–h). This idea is further supported as 180° bi-polar cells are less frequent in PreMegE, Mkps, PreCFU-E, CFU-E, and Pro-Ery progenitors compared to HSCs and MPP1–5s (Extended Data Figs. 6e–h). Together, these data show that the relative positioning of two poles within a cell is not random and may influence cell function.

To understand what changes in the relative position of poles might mean, we next asked if the number and orientation of Tubulin, CDC42-total, phospho-CDC42, and/or Actin poles change as cells progress through the cell cycle. While cells with 180° Tubulin poles might represent duplicated centrosomes that moved to the opposite pole before division, cells with single poles could be in G1, and cells with poles at 90° or 270° in S/G2, like cell cycle-mediated CDC42 polarity changes in yeast.^17^

**Extended Data Fig. 8.**
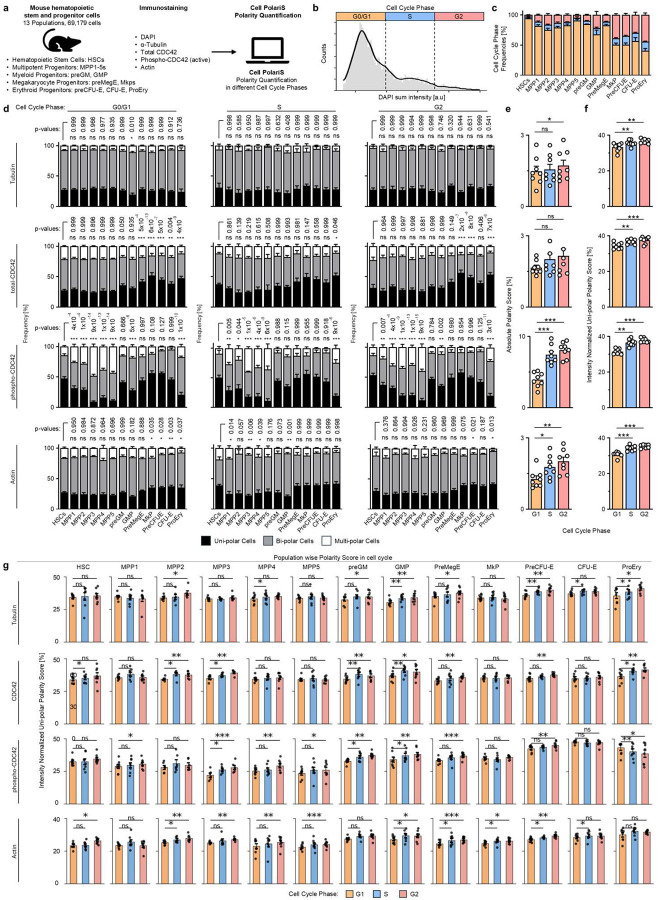
Cell polarity increases throughout the cell cycle. **a,** Experimental design. 13 freshly isolated hematopoietic stem and progenitor populations were fixed and stained for DAPI, a-Tubulin, total-CDC42, phosphor-CDC42, and Actin to determine whether cell polarity changes throughout the cell cycle. **b,** Representative DAPI intensity distribution used to classify freshly isolated HSPCs into G0/G1, S, and G2 cell cycle phases. **c,** Relative frequencies of freshly isolated HSPCs in G1, S, and G2, respectively. As previously reported, most HSCs and MPP1–5 are in G0/1, while actively cycling later progenitors are more often found in S and G2. **d,** Relative frequencies of uni-polar, bi-polar, and multipolar cells in G0/G1, S, and G2 for Tubulin, total-CDC42, phospho-CDC42, and Actin. The number of cell poles does not change throughout the cell cycle. **e and f,** Quantification of absolute and intensity normalized polarity for Tubulin, total-CDC42, phospho-CDC42, and Actin for cells in G0/G1, S, and G2 cell cycle phases. As most cell growth with cell cycle progression increases in the absolute polarity score are expected and result from cell cycle-related increases in marker expression levels. However, the intensity-normalized polarity score also shows that polarity increases from G1 to S and G2, suggesting that cell polarity increases through cell cycle progression. **g,** Quantification of intensity-normalized polarity for Tubulin, total-CDC42, phospho-CDC42, and Actin for each HSPC population in G0/G1, S, and G2 cell cycle phases, respectively. P values were calculated by two-way ANOVA with Tukey’s multiple-comparisons test for panels in d and one-way ANOVA with Tukey’s multiple-comparisons test for panels in e-g; ns, not significant (P > 0.05); *P < 0.05; **P < 0.01; ***P < 0.001.

This means that changes in polarity between HSPCs might merely reflect changes in G1, S, and G2 frequencies between slow and fast cycling populations. Indeed, HSCs and MPP1–5s are more often found in G0/G1 compared to later progenitors (Extended Data Figs. 8a–c). Using DAPI, we classified cells into G1, S, and G2 to test if polarity changes throughout the cell cycle. Although we find that the number of cell poles is constant throughout the cell cycle (Extended Data Fig. 8d), polarity scores increase slightly from G1 to G2 (Extended Data Figs. 8e–f) in some cell types, these changes are too small to explain why polarity differs between HSCs, MPP1–5s, and erythroid progenitors for phospho-CDC42 and Actin (Figs. 4i,j; Extended Data Figs. 6k,l).

#### Cell polarity is highly dynamic and can change within minutes.

**Fig. 5.**
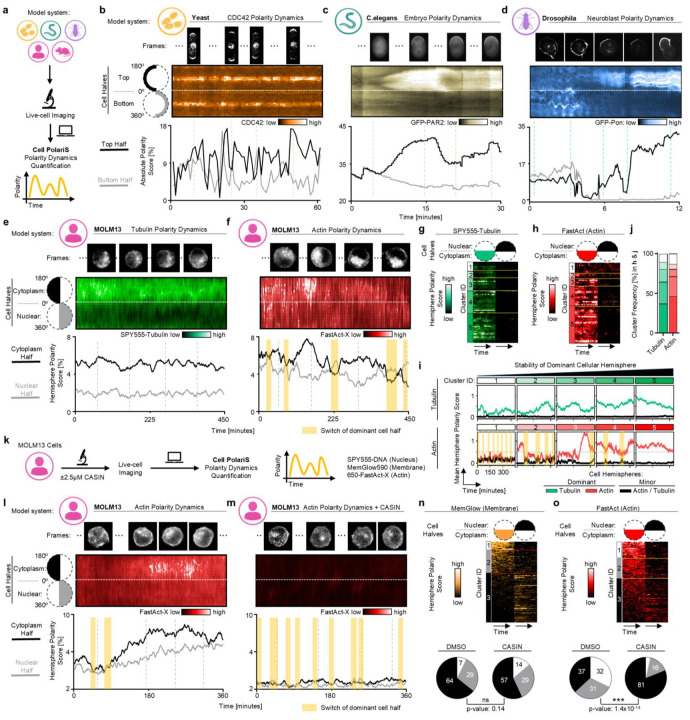
Live cell polarity dynamics discovers both complex and multipolar cell behavior. **a**, Experimental Design. Polarity dynamics analysis of live-cell imaging data from fission yeast, the C. elegans embryo, Drosophila Sensory Organ Precursor (SOP), and human MOLM13 cells using CellPolariS with multiple fluorescent reporters, including CRIB-mCherry (CDC42), GFP-PAR2, GFP-PON, SPY555-Tubulin, and FastAct-650. **b-d**, Quantification of cell polarity over time in fission yeast (b), C. elegans embryo (c), Drosophila Sensory Organ Precursor (SOP) (d) labeled with active CDC42, GFP-PAR2, and GFP-Pon, respectively. Top: Selected frames to illustrate redistribution of CDC42, GFP-PAR2, and GFP-PON, from one cell pole to the other, respectively. Middle: Kymograph showing Active CDC42, GFP-PAR2, and GFP-Pon redistribution 360 degrees over time from one top cell pole to the bottom cell pole. Bottom: Active CDC42, GFP-PAR2, and GFP-Pon quantification in the top (black line) and bottom (grey line) half of the cells over time. CDC42 polarity is dynamic, with CDC42 reversibly redistributing from one cell pole to the other (b). In the C. elegans embryo, GFP-PAR2 polarity is gradually established before asymmetric division. The Drosophila SOP has multiple GFP-Pon poles before division, which fluctuate and reorganize to a main pole on the opposite side of the cell before asymmetric division. Cell polarity is a highly dynamic process and can change within minutes in non-dividing and dividing cells. **e-f**, Representative example of Tubulin (e) and Actin (f) polarity dynamics quantification. Both Tubulin and Actin have a primary, dominant cell pole (top half). While the primary Tubulin pole is stable over time, the primary (top half) and secondary (bottom half) Actin poles compete. This means that when the polarity of the primary pole drops, the polarity of the secondary pole rises (highlighted orange boxes). **g-j**, Hierarchical clustering of Tubulin and Actin polarity dynamics shows that most cells have a dominant and stable tubulin cell pole, while actin poles and polarity dynamics. High F-actin levels (above dashed line) appear to support the dominance of the primary cell poles, while when F-Actin levels drop, a secondary cell pole can form. **k**, Experimental design. MOLM13 cells were stained for SPY555-DNA (Nucleus), MemGlow590 (Membrane), and 650-FastAct-X (F-Actin), cultured ±2.5μm CASIN (CDC42 inhibitor), and imaged continuously for 5 hours every 30 seconds to determine if CellPolariS can detect the expected drop in Actin polarity after inhibition of the pro-polarity factor CDC42. **l, m,** Quantification of cell polarity over time in MOLM13 cells labeled with SPY555-DNA, Glow590, and 650-FastActin-X to label the Nucleus, Membrane, and Actin, respectively. Top: Selected frames to illustrate the redistribution of Actin from one cell pole to the other. Middle: Kymograph showing Actin redistribution 360 degrees over time from one top cell pole to the bottom cell pole. Bottom: Actin quantification in the top (black line) and bottom (grey line) half of the cells over time. Actin polarity is dynamic, with Actin reversibly redistributing from one cell pole to the other. Cell polarity is a highly dynamic process and can change within minutes in non-dividing and dividing cells. **m**, **o,** Representative Actin polarity dynamics quantification in DMSO (l) and CASIN (m). While in DMSO, F-Actin levels and polarity are higher in the primary pole (top half), CDC42 inhibition reduces overall actin polarity. However, although at a lower level, the primary pole (top half) remains dominant and more polarized. **n,** Hierarchical clustering of MemGlow and FactAct polarity dynamics shows that CDC42 inhibition with CASIN reduces F-actin levels and polarity as expected, while the membrane polarity remains unchanged. P values were calculated by Pearson’s χ^2^ test of independence for the global comparison in each panel, followed by post-hoc two-proportion z-tests with Holm–Bonferroni correction across the three categories; ns, not significant (P > 0.05); *P < 0.05; **P < 0.01; ***P < 0.001.

Even highly purified cells in G1, S, and G2 cell cycle phases remain heterogeneous in terms of polarity (Extended Data Fig. 8d), suggesting either (1) the existence of additional subpopulations or (2) that polarity is more dynamic than previously appreciated. To investigate this, we extended CellPolariS to also quantify polarity dynamics using time-lapse data and validated our approach by recapitulating previously studied CDC42, PAR2, and PONS polarity dynamics in fission yeast and during asymmetric divisions of C. elegans and D. melanogaster, respectively (Figs. 5a–d). Although these movies are relatively short, cells often move and change orientation over time, which can alter the quantification of the direction and relative position of multiple fluorescent markers to each other over time (Extended Data Fig. 3a). CellPolariS therefore corrects and aligns the cellular orientation based on a cellular reference marker (e.g., nucleus), to ensure that even when cells turn around to reorient themselves, the relative position of fluorescent signals over time are conserved.

**Extended Data Fig. 9.**
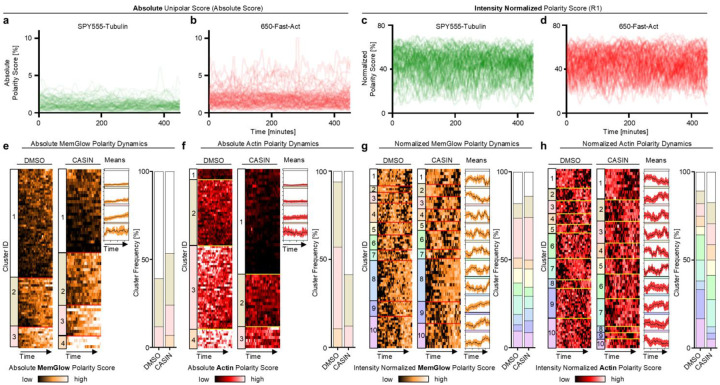
CASIN reduces Actin polarity with and without intensity correction. **a-d,** Absolute and intensity-normalized Tubulin and Actin polarity score dynamics over time. Cell polarity is heterogeneous between cells and changes dynamically over time. 76 time series analyzed FastAct and Tubulin, respectively. **e-h,** Absolute and intensity-normalized membrane (MemGlow) and Actin (FastAct) polarity dynamics ±CASIN (CDC42 inhibitor). CDC42 inhibition reduces both absolute and intensity-normalized Actin polarity and its dynamics over time, without affecting membrane polarity. 142 time series analyzed in total, including 84 for DMSO and 58 for CASIN, respectively.

Using this correction, we next investigated polarity dynamics and heterogeneity of human MOLM13 cells, which we stained for Tubulin and Actin and imaged every 30 seconds for 4 hours. We find that Tubulin and Actin polarity can vary widely among cells (Extended Data Figs. 9a–d), and that the cytoplasmic cell half dominates compared to the nuclear half (Figs. 5e–i). Furthermore, while for Tubulin the cytoplasmic cell half retains dominance for 4 hours, Actin polarity is more dynamic (Figs. 5e,f,i) and the nuclear half gains dominance when Actin levels drop (Figure 5i, dashed line and orange indicators). This analysis also showed that Actin polarity can change within minutes from a polar to an apolar state (Extended Data Figs. 9b,d). To investigate this further, we next stained MOLM13 with SPY555-DNA, 650-FastActX (Actin), and MemGlow590 (Membrane), and imaged the cells treated with ±2.5μm CASIN (CDC42 and polarity inhibitor) (Fig. 5k). As expected, CASIN reduces Actin levels and polarity, but does not affect the membrane polarity (Figs. 5l–o, Extended Data Figs. 9e–f). In addition, and in line with our hypothesis, we find that when Actin levels on the dominant cell half drop, Actin can distribute and polarize transiently on the nuclear cell half (Figs. 5l,m; orange indicators). Together, this shows that CellPolariS can, in addition to analyzing fixed cells, robustly quantify polarity dynamics in videos.

## Discussion

Here, we present CellPolariS, the first fully automated tool capable of quantifying polarity in images of any fixed or living cell with high sensitivity and at scale. Beyond providing a novel method to quantify polarity, CellPolariS overcomes key limitations of existing tools, including low accuracy, sensitivity, throughput, and/or restrictions to specific cell types. Using extensive validation, including (1) over a million simulated cells, (2) 86 genetic perturbations in 112,366 T-cells, and (3) 13 different cell types and multiple species. Our tool thus enables systematic large-scale polarity studies, making CellPolariS broadly applicable to study fundamental and clinically relevant cellular properties across different fields and diseases.

These novel capabilities arise from a fundamentally different approach that, rather than assuming cells are round, transforms cells into circular discs for direct, fair quantitative comparison. In contrast to existing approaches, which reduce the complex distribution of cellular constituents into a single score, we leverage the spatial information in fluorescence images to detect the number, strength, and direction of marker accumulation, thereby improving accuracy and sensitivity. CellPolariS works robustly irrespective of cell size, shape, and marker expression, as validated with different cell types, including bacteria, yeast, T-Cells, natural killer cells, and neurons, and can be easily adapted by others.

We also provide new insights and show that polarity (1) changes during hematopoietic differentiation from HSCs towards MPPs and committed progenitors, (2) changes throughout the cell cycle, and (3) is highly dynamic, as cells can switch between polar and apolar states within minutes. Unlike previous studies, which relied on the analysis of snapshots alone, assuming that polarity is a stable cellular property, our findings show that polarity is dynamic. Importantly, our novel insights suggest that previously reported differences in polarity between different cell populations and/or culture conditions might actually have been caused by confounding factors rather than changes in polarity itself and should therefore be reevaluated.

Despite the novel capabilities, CellPolariS has practical limitations that we plan to address in the future. First, like other approaches, CellPolariS’ accuracy depends on high-quality image segmentation, which can be challenging depending on cell density, cell types, and marker distribution. However, recent advances in artificial intelligence have created off-the-shelf advanced segmentation models, including Segment Anything (SAM)^18^, Cellpose-SAM^19^, and Self-supervised Learning (SSL)^20^, which enable non-experts to obtain accurate segmentation. Second, in its current implementation, CellPolariS analyzes polarity in two dimensions, which may affect the quantification of polarity in a small subset of cells. Future studies extending CellPolariS to quantify polarity in 3-dimensions will be important to quantify polarity in complex in-situ microenvironments and organoids.

Our novel approach has important implications for studying disease mechanisms in a wide variety of tissues. As CellPolariS is the first tool to correct cell morphology, it can be applied to images of any cells or tissue, and its unprecedented throughput and sensitivity enable the community to detect polarity changes that would otherwise be missed. By developing a novel way to quantify cell polarity with unprecedented accuracy and throughput, CellPolariS enables the study of the molecular regulation and dynamics of cell polarity, one of the most fundamental yet poorly understood biological processes with clinical relevance.

## Online Methods

### Research animals

All experiments were conducted with 12–18-week-old C57BL/6J mice purchased from The Jackson Laboratory (#000664) and acclimatized in St. Jude’s Animal Research Center (ARC) for at least 1 week before the start of an experiment. Mice were housed in hygienic conditions in individually ventilated cages with 2–5 mice per cage and supplied with environmental enrichment. Mice were housed with an inverse 12 h day–night cycle in a temperature (21 ± 2 °C) and humidity (55 ± 10%) controlled room with ad libitum access to standard diet and always drinking water. The general well-being of the mice was routinely monitored by animal caretakers through daily visual inspections. Mice were euthanized if symptoms of pain and/or distress were observed. Mice were randomly assigned to experimental groups and pooled for experiments to reduce biological variability.

### Cell Lines

Human MOLM-13 cells were a gift from Dr. Charles Mullighan, St. Jude Children’s Research Hospital, authenticated using Short Tandem Repeat (STR) analysis, and cultured in RPMI supplemented with 10% horse serum (HS), 1% Pen-Strep (50 U/mL penicillin and 50 μg/mL streptomycin, #2827307, Gibco) at 37 °C, 21% O_2_, and 5% CO_2_. After thawing, cells were passaged every 2 to 4 days and cultured for three passages before experiments.

### Primary hematopoietic stem- and progenitor-cell isolation

Mouse primary cells were isolated and sorted as described^2,21^. Briefly, tibiae, femurs, coxae, humeri, ulnae, and vertebrae were crushed in FACS buffer (PBS, 2% HS, 2 mM EDTA) and filtered using a 100-μm nylon mesh. Erythrocytes were lysed with ACK lysing buffer (Gibco) for 5 min on ice. Cells were then stained with biotinylated lineage antibodies against CD19 (clone: MB19–1), CD3ε (145–2C11), TER-119 (TER-119), B220 (RA3–6B2), Ly-6G (RB6–8C5), and CD11b (M1/70), followed by labeling with streptavidin-conjugated magnetic beads (50001, Stem Cell Technologies). Following immuno-magnetic depletion, cells were stained with CD150–BV650 (#115932) or BV711(#115941; clone TC15–12F12.2), CD48–FITC (#103404; clone HM48–1), cKIT–PE–Cy7 (#105814) or APC/Fire 750 (#105838; clone 2B8), SCA1–PerCP–Cy5.5 (#108124) or BV785 (#B409405; clone D7) (all Biolegend), CD135–PE–CF594 (#562537; clone A2F10.1; BD), CD34-e660 (#50–0341-82; clone RAM34; Invitrogen), streptavidin–e450 (#48–4317-82; Invitrogen) overnight on ice.

### Fluorescence-activated cell sorting

All cells were sorted directly into a sterile 1.5 mL Eppendorf tube containing medium using a BD FACS Aria Fusion or FACSymphony S6 with a 70-μm nozzle, single-cell purity mode, and sorting purities ≥ 98% determined by pre- and post-sort purity controls (see supplemental Table 2 for optical configuration). All sorts were done using PBS as the sheath fluid. Before all sorts of machines were cleaned for 10 minutes with 10% bleach and water, calibrated using Accudrop beads, and aligned. OneComp eBeads (#01–1111-42, ThermoFisher) were used for compensation, and dead cells were excluded based on NucBlue (#R37606, Invitrogen) staining. Debris and doublets were excluded based on gating on forward (FSC) and side scatter (SSC) (see supplemental Fig. 1).

### Immunofluorescence staining

Freshly isolated cells were fixed on μ-Slide VI^0.4^ slides (#80606, Ibidi GmbH) coated for 1 hour with 20μg/mL α-CD43-biotin (#13–0431-85, ThermoFisher; clone eBioR2/60) as described^22^. Cells were then added to slides and allowed to settle for 30 minutes at 37 °C, 21% O_2_, 5% CO_2_ before fixation with Image-iT 4% paraformaldehyde in PBS (Immunogen, #I28800), permeabilization with 0.5% Triton-X 100 (Sigma-Aldrich), and blocking with Intercept Blocking Buffer (#927–70001, Li-Cor inc.) for 1 hour at room temperature, then incubated with the primary conjugated antibodies against, phospho-CDC42(Ser71)-Alexa Fluor 555 at 0.5μg/mL (#bs-3369R-555, Bioss antibodies), CDC42-Alexa Fluor 647 at 0.5μg/mL (#ab215234, Abcam), β-Tubulin-Alexa Fluor 488 at 0.5μg/mL (#bsm-33034M-A488, Bioss antibodies) in blocking buffer at 4° C overnight. Samples were then washed 3x with wash buffer (minimum 5-minute incubation for each wash) and stained with DAPI (1μg/mL in PBS) for 10 minutes at room temperature, followed by 3 washes before imaging in PBS. Cells were imaged within 72 hours after staining. List all antibodies used in this study in a .csv file.

Images were acquired with a Nikon Ti-2 widefield microscope using NIS-Elements AR (version 5.42.04), and a Plan Apochromat λ 20x objective (NA 0.75), 16x averaging, 16-bit pixel depth, and through a Z-depth of 16 microns and nine 2.0 μm steps, which is below the Nyquist minimum recommended step size of 2.4 μm.

### Microscope Image Hardware and Image Acquisition Settings

Experiments were conducted using a Nikon-Ti2 Eclipse equipped with a linear encoded motorized stage, a 10.2 Megapixel Photometrics Kinetix Camera with 6.5μm × 6.5μm pixel size (Teledyne), and a Spectra III fluorescent light source (solid-state light sources at 365nn (395/25nm), 440nm (438/29nm), 488nm (475/28nm), 514nm (511/16nm), 561nm (555/28nm), 594nm (575/25nm), 635nm (637/9nm), 650nm (661/20nm) and 730nm (740/20nm) (Lumencor) and Nikon White light LED for transmitted light bright field illumination. Experiments were done at 37°C (Okolab Heating unit, H201-T-UNIT-BL), 5% O_2_, 5% CO_2_, using either a manual gas mixer (Okolab 3GF-Mixer-Hypoxia) or the environmental control unit (OkoLab Bold Line Gas Mixer), custom 3D printed stage top incubators, and a humidifier to prevent evaporation. Fluorescent images were acquired using optimized filter sets: Violet (395/25; 420; 435/26), CFP (436/20; 455LP; 532/18), eGFP (470/40; 495LP; 525/50), a555 (546/10; 555; 575/25), mCherry (550/32; 585LP; 605/15), a594 (598/25; 605LP; 642/10), Cy5 (620/60; 660LP; 700/75; all AHF) to detect DAPI, BV480, AlexaFluor488, SPY-555 DNA, MemGlow590, BioTracker 650 Red Nuclear dye/SPY650 FastAct-X, respectively (see Supplementary Table 3 for more details). Time intervals of bright field and fluorescent image acquisition were chosen to minimize phototoxicity. Images were acquired using a 10 × CFI Plan Apochromat λ objective (NA 0.45). Single-cell tracking and image quantification were performed using self-written software as described^24–28^.

### Immunostaining Image quantification and analyses

Acquired 16-bit images with 3200 × 3200-pixel resolution were saved as. nd2 files, corrected for uneven illumination in NIS-Elements using the Shading Correction function with Background smoothness = 10 and Black Background (Fluorescence) mode, and exported as .tiff.

### Time-lapse Image quantification and analyses

Images were acquired with NIS-Elements AR (version 5.30.06) as 16-bit with 3200 × 3200-pixel resolution, saved as. nd2 files and exported to .tif for further processing. 16-bit images were then linearly transformed to 8-bit using channel-optimized white points, background correction using BaSiC^23^, and brightfield channel segmentation using FastER^26^. Trained labeling masks were dilated (settings: dilation 6) to ensure proper segmentation and quantification of the entire cell in all fluorescence channels. Tracking and quantification of fluorescence channels of single cells over time were done as described and analyzed using MATLAB 2023b (MathWorks)^26^.

Human MOLM13 cells were cultured on μ-Slide VI^0.4^ channel slides coated with 10μg/mL anti-CD43/Sialophorin (MEM-59) biotin (#NBP2–62227B, Novus Biologicals) in PBS for 1 hour^22^ in phenol-red-free IMDM media (#2365797, Gibco) supplemented with 20% BIT Serum substitute (#1000073632, STEMCELL Technologies) and 1% Pen-Strep (50 U/mL penicillin and 50 μg/mL streptomycin, #2827307, Gibco) and allowed to settle onto the coated slides for at least 30 minutes at 37 °C, 21% O_2,_ and 5% CO_2_. For visualization of cell lines, cells were incubated with the live cell dyes BioTracker 650 Red Nuclear Dye at 0.5x concentration (1:2000 dilution) (#SCT119, Sigma-Aldrich), SPY650-FastActX at 1x concentration (1:1000 dilution) (#CY-SC205, Cytoskeleton Inc.), SPY555-DNA at 1x concentration (1:1000 dilution) (#CY-SC201, Cytoskeleton Inc.), and MemGlow590 at 0.04 μM (1:500 dilution) (#MG03, Cytoskeleton Inc).

Cells were incubated with 2.5μM CASIN (HY-12874, MedChemExpress) to perturb polarity and actin dynamics without fully abolishing F-actin activity, then compared to DMSO-incubated controls. Cell line time-lapse images were acquired with a Plan Apochromat λ 20x objective (NA 0.75) in a single Z plane.

Time-lapse images were acquired with the following parameters:

BioTracker 650 Red Nuclear Dye: LED power: 200mW, LED Power: 2.5%, Exposure time: 100ms. Filter cube: Ex: 620/30, Dichroic: 645, Em: 660/13, Binning: 1×1, imaged every 60s.
SPY650-FastActX: LED power: 200mW, LED Power: 3.0%, Exposure time: 200ms. Filter cube: Ex: 620/30, Dichroic: 645, Em: 660/13, Binning: 1×1, imaged every 30s.
MemGlow 590: LED power: 500mW, LED Power: 15.0%, Exposure time: 400ms, Filter cube: Ex: 598/25, Dichroic: 605, Em: 642/10, Binning: 1×1, imaged every 30s,
SPY555-DNA: Light source power: 500mW, LED Power: 17.0%, exposure time: 300ms, Filter cube: Ex: 546/10, Dichroic: 555, Em: 575/25, Binning: 1×1, imaged every 30s.

### Datasets

#### Generation of synthetic cells for systematic analysis of confounding factors:

To benchmark Cell Polaris’s ability to classify uni, bi-, and multipolar cells, we generated 30,000 synthetic circular cells (radius 60 pixels) with a uniform background (pixel intensity: 4,000) and 1–3 randomly positioned fluorescent dots (radius 15 pixels; pixel intensity 35,000) using custom Python code (Figs. 1k–m).

To systematically assess and quantify how circular morphological transformation preserves and/or alters key cell features (Fig. 2e), we generated 1,000,000 synthetic cells with increasingly complex morphologies, representing the following five major cell shapes: ellipsoid, amoeboid, filamentous, neurite-like, and stellate (200,000 cells per class) using custom Python code. To ^13^simulate elongated, curved, and progressively branched cells found in nature, we used dots, lines, and curves as seeds, which were then expanded by iterative pixel addition.

#### Biological data set used for real-world validation:

To understand how irregular cell shapes can displace the geometric center from the ‘True’ cell center and affect cell polarity quantification in real cells, we use data from *C.elegans* embryo^24^, Jurkat T-cell^13^, Natural Killer cell^13^, *E.coli*^24^, Motor neuron^25^, and HUVEC cells^26^.

To validate that CellPolariS also works reliably with images of real cells, we reanalyzed publicly available high-content imaging data of human T and NK lymphocytes^13^ downloaded from Image Data Resource (Fig. 3). We downloaded datasets corresponding to activated/stimulated Jurkat T cells on ICAM and poly-L-Lysine-coated surfaces (PLL). For each plate, well, field, and channel, we grouped all available z-planes and generated a maximum-intensity projection, which we saved as a TIFF image for downstream analysis. We excluded empty or corrupted image files before projection. Associated plate layouts and quantitative measurements were obtained from the linked Fighare resource, and well annotations were retrieved from the corresponding GitHub repository supplied with the study.

For live-cell imaging analyses, we used published movies from C. elegans^27^, yeast cells^28^, and D. melanogaster sensory organ precursor^29^. For the C. elegans and D. melanogaster SOP datasets, we downloaded MP4 videos and extracted grayscale TIFF images corresponding to the relevant marker.

### Image processing - Extended-depth-of-field (EDF)

To preserve fluorescent signals from different z-slices and remove out-of-focus light in widefield images, we converted the z-stacks into Extended-depth-of-field images prior to analysis using a Python implementation of the selective fusion framework as described^30^. For this, we aligned z-stacks to the middle slice by correlation coefficient registration (maximum 200 iterations; convergence threshold 10^−6^). Background was estimated independently for each plane by Gaussian smoothing (σ=120 pixels) and subtracted. Residual vertical striping was reduced by subtracting 0.4 × a smoothed column-median profile (σ=120 pixels). For focus estimation, each plane was scaled to its 99th intensity percentile. Local focus was then defined as the intensity variance within an 11×11 pixel neighborhood. At each pixel, we then normalized focus scores across z to generate fusion weights, which were further modulated by an intensity gate derived from a Gaussian-smoothed image (σ=1.5 pixels; exponent 0.7) and spatially smoothed (σ=1.0 pixels). We then computed a soft depth map as the weighted mean z-position at each pixel and regularized by guided filtering (radius = 25 pixels, ε=0.01) using the maximum projection of the background-corrected stack as the guide image. We then recalculated, for each pixel, the contribution of each z-plane to the final EDF image by centering a Gaussian weighting function on the regularized depth estimate ($\sigma_{z}\;=\;1.0$ z-step). To avoid mixing signals from many out-of-focus planes, only the two most strongly weighted z-planes were retained at each pixel (k=2; exponent 1.5), and the final all-in-focus image was generated as their weighted sum. A mild unsharp mask was applied to the fused image by subtracting a Gaussian-blurred version of the image (σ=1.0 pixel) and adding back the residual with a strength of 0.45. To suppress residual background offset, a low-intensity floor corresponding to the 1st percentile of the image intensity distribution was subtracted, and negative values were clipped to zero. Final EDF images were then clipped to the 16-bit dynamic range [0, 65535] and exported as 16-bit TIFF files.

### Cell Polarity Quantification using CellPolariS

As illustrated in Extended Data Figure 2, Polarity quantification using CellPolariS relies on nine sequential steps. In **Step 1**, cells are detected using previously published segmentation tools. In **Steps 2 and 3,** CellPolariS calculates the morphological cell center as reliable reference points for later polarity quantifications and corrects for irregular cell shapes, which can prevent reliable polarity quantification. In **Step 4**, CellPolariS quantifies the polarized spatial distribution of fluorescence signals by calculating the sum pixel intensities in 60 6-degree-wide (adjustable) slices, creating polarity vectors that preserve the direction (angle), height, width, number, and relative positions of fluorescence peaks. In **Steps 5, 6, and 7,** several confounding factors, including cell size differences, marker intensity variation, and platform dependent to ensure images acquired with different microscopes, cameras, and magnifications are comparable across studies. Finally, in **Step 8**, CellPolariS corrects for variation in cellular orientation and quantifies polarity in **Step 9** using unsupervised clustering to classify cells into apolar and polar cells with one, two, or multiple poles.

### Step 1: Segmentation

Although compatible with any segmentation method, CellPolariS can detect cells using state-of-the-art segmentation methods, including StarDist^31^, Cellpose-SAM^32^, and classical Otsu thresholding, providing flexibility for advanced users and ease of use for image analysis novices.

### Step 2: Identification of the morphological cell center as a reliable reference point

As cell polarity quantifications often rely on the cell center as a reference point, identifying the ‘actual’ cell center correctly is critical. However, in irregularly shaped cells, the geometric cell center (the reference point) and the biologically meaningful cell center often do not overlap, which can alter polarity quantifications (see Fig. 2f). As most cells are not perfectly round, correcting for irregular cell shapes is a prerequisite for reliable polarity quantification, but has thus far been ignored as a critical step for polarity quantification.

To address this, we developed a shape-adaptive reference point calculation, which derives the morphological cell center using the shape of the segmented cell mask. Unlike the geometric cell center, which relies on simple geometric averaging, our approach iteratively erodes the outer pixel layers of cell masks until only a single residual pixel, or a minimal residual region, remains. This residual point (the morphological cell center) reliably detects the biologically meaningful cell center in both round and irregularly shaped cells, even in cells extreme irregular cell morphologies, such as neurons. The morphological cell center can therefore serve as a common, biologically meaningful reference point for quantifying polarity across cells with vastly different morphologies.

### Step 3: Correcting for irregular cell shapes using a perfect one-to-one pixel mapping circular transformation algorithm

Using the morphological cell center as a reliable cellular reference point, we next developed a circular transformation algorithm that converts cells, irrespective of their morphology, to a common circular reference geometry, enabling a fair comparison between cells with different morphologies. Conceptually, our approach is analogous to peeling an onion layer by layer and reorganizing those layers into concentric rings. Importantly, our approach maintains the precise pixel-level information of the original cells, as the total number of pixels, their pixel intensities, and their order during mapping in concentric rings are identical. While this approach also preserves the axial sequence of pixels (center to cell edge), the precise angles between pixels in adjacent concentric rings might be distorted slightly, as with any other transformation that is used, for instance, creating a world map.

Specifically, during the transformation, individual pixels are mapped one-to-one, from the original segmented cell mask to a circularized representation, such that each cellular pixel is assigned to one unique position in the transformed image. For this, the cell masks are decomposed into successive one-pixel-thick layers by iterative morphological erosion. The outermost layer is ordered by contour tracing and aligned to a reference anchor angle defined from the major axis of the cell mask by principal component analysis of mask-pixel coordinates relative to the morphological center. Each subsequent inner layer is then ordered relative to its parent layers using angle-constrained parent tracking, thereby preserving angular correspondence across neighboring layers as the procedure progresses inward toward the morphological center.

The concentric ring radii and pixel numbers are selected to accommodate the number of pixels in each layer while preventing overlaps between adjacent rings. Pixel intensities from the original image were then transferred directly to their assigned circular coordinates, yielding a transformed image in which every source pixel is represented exactly once.

### Stage 4: Polarity vector calculation

As classical polarity scores reduce the subcellular spatial distribution of fluorescence signals into a single number, these scores cannot detect the number, magnitude, and relative position of cell poles, and therefore lead to the incorrect classification of polar vs. apolar cells. To overcome these limitations and issues, CellPolariS quantifies the sum pixel intensities of 60, 6-degree wide (Δθ) wedges (one wedge spans 6 degrees of a 360-degree circle) to preserve information about the spatial distribution, orientation, magnitude, width, and number of fluorescent signals that are otherwise lost using other methods. Contrary to classical polarity scores, the default polarity vector contains 60 wedges. Importantly, to maximize flexibility, the number of wedges per cell can be adjusted to, for instance, 72 5-degree wedges if needed.

For wedge i, we compute the mean signal intensity $w_{i}$ at the angular position $\theta_{i}$, yielding the polarity vector $w=\left(w_{0},\ldots ,w_{K-1}\right)$, where K is the total number of wedges.

### Step 5: Cell-size normalization

Cell size variation between different cell types or cell cycle phases can impact polarity quantification, as the ‘weight’ of the fluorescence signal is higher when the distance to the cell center increases. This means that two otherwise identical cells (same intensity distributions and overall proportions) can incorrectly appear to differ in polarity if one cell is slightly larger or smaller. To correct for this effect, which is often ignored in simple polarity quantification methods, such as the ’Delta Centroid Method’ (see Fig. 1b), CellPolariS normalizes for cell size variation by calculating the mean pixel intensities of 60 slices (default setting: 6-degree spacing).

### Step 6: Intensity normalization

As individual cells of highly purified cell populations can differ several orders of magnitude in marker expression and intensity, among two cells with the otherwise same fluorescence signal distribution (at identical proportions), the cell with higher expression will always have a higher polarity score (see Extended Data Figure 2b–d). Importantly, this also means that cells with high marker expression might appear incorrectly to be more polarized compared to their low-expressing counterparts. To correct this confounding factor, CellPolariS can normalize the polarity vectors using the following methods:

1. **Min–max range normalization** per wedge $\left(w_{i}^{\left(\text{mm}\right)}\right)$, which is calculated as follows,
$$w_{i}^{\left(\text{mm}\right)}=\frac{w_{i}-\text{min}(w)}{\text{max}(w)-\text{min}(w)}$$
  $w_{i}:$ *Mean intensity of wedge* i
  w: *Polarity vector* $\left(w_{0},w_{1},\ldots ,w_{K-1}\right)$
  min(w): *minimum wedge intensity within a cell*
  $max\left(w\right):$ *maximum wedge intensity within a cell*
2. **Z-score normalization** per wedge ($w_{i}^{\left(\text{z}\right)}$), which is calculated as shown below,
$$w_{i}^{\left(z\right)}=\frac{w_{i}-\overset{‾}{w}}{\sigma_{w}}$$
  With
$$\overset{‾}{w}=\frac{1}{K}\sum_{i=0}^{K-1}w_{i},\;\sigma_{w}=\sqrt{\frac{1}{K}\sum_{i=0}^{K-1}{\left(w_{i}-\overset{‾}{w}\right)}^{2}}$$
  $w_{i}:$ *Mean intensity of wedge* i
  $\overset{‾}{w}:$ *Mean intensity wedges of the cell*
  $\sigma_{w}:$ *Minimum wedge intensity within a cell*
  K: *Total number of wedges*.
3. **Percentile-based normalization** per wedge $\left({w_{i}}^{\left(\text{pr}\right)}\right)$
$$w_{i}^{\left(\text{pr}\right)}=\frac{w_{i}-P_{2}(w)}{P_{98}(w)-P_{2}(w)}$$
  wi: *Mean intensity of wedge* i
  w: *Polarity vector* $\left(w_{0},w_{1},\ldots ,w_{K-1}\right)$
  P2(w): *2nd percentile of wedge intensities within that cell*
  *P*98(w): *98th percentile of wedge intensities within that cell*.

### Step 7: Image Normalization using synthetic reference cells

To ensure polarity quantifications are comparable between studies that acquired images using different hardware and software settings, CellPolariS standardizes each cell using two synthetic reference cells that represent a completely apolar cell and a cell with maximum polarity (see Fig. 1b). This means, for example, if 16-bit images are used for analysis, CellPolariS automatically uses a polar reference cell in which one half of the cell has the maximum possible pixel intensity of 65535, while all pixels in the other cell half have the pixel intensity 1. In 8-bit images for comparison, the pixel intensities of the reference cell have the values 255 and 1 on either side. This standardization ensures that (1) polarity calculations are comparable even when image acquisition settings change, and (2) makes calculated polarity values intuitively comparable between samples and different fluorescent channels because CellPolariS polarity scales between 0 (minimum) and 100% (maximum). These rescaling steps are calculated as shown below:

$$\text{Rescaled}\;\text{Polarity}\;R_{m}\left(\%\right)=100\times \frac{\text{Polarity}\;\text{magnitude}\;V_{m}}{\text{Polarity}\;\;{\text{magnitude}}_{m}^{\text{ref}}}$$

m: Number of poles (m=1: uni-polar, m=2: bipolar organization)

### Step 8: Correcting for different cell orientation using cross-correlation

For technical (e.g., camera rotation) and biological reasons (e.g., cell motility), even cells with identical fluorescence distribution can orient their cell bodies in different directions, making direct comparison and quantification of similarities between angular fluorescent intensities difficult. To correct cell orientation, CellPolariS aligns all cellular polarity vectors to a common synthetic Gaussian reference distribution by calculating their offset using circular cross-correlation. Offsets were computed by FFT-based convolution and applied as circular shifts, yielding reference-aligned profiles on a common angular frame.

### Step 9: Cell Polarity Quantification and Classification using unsupervised clustering

To analyze cell polarity, CellPolariS then classifies the fully corrected polarity vectors using unsupervised hierarchical agglomerative clustering with Ward linkage and Euclidean distance. While the number of clusters can be adjusted and determined by the user, we found that 5 clusters provided a reasonable separation between different polarity classes. CellPolariS then visualizes the results as heat maps and calculates the absolute and relative frequencies and exports Polarity Vectors along with Classical Polarity Scores, fluorescence, and morphology-based cell features (e.g., Sum Pixel Intensities, Area, Perimeter) as .csv.

### Validation and Quality Control Metrics

To verify that the morphological transformation preserves key cell features, we quantified the total number of pixels, the pixel intensities, and the local pixel organization before and after morphological transformation (see Fig. 2e). For this, we quantified the inner-neighbor preservation between original and transformed cells based on 8-connected pixel neighborhoods. We calculated the degree of tangential preservation by computing the circular distance of neighboring pixels within the same layer before and after transformation, and considered pixel distance to be preserved when the distance was equal to the original distance. Radial preservation was evaluated for neighboring pixels assigned to different layers by comparing their relative angular positions across layers.

### CellPolariS Polarity Score Calculations and Interpretation

Although CellPolariS’s polarity vector is more accurate and reliable than simple polarity scores, CellPolariS can calculate multiple scores for different purposes, including the (1) uni-polar, (2) bipolar, and (3) hemisphere polarity scores. For polarity score calculation, the circularized cell was partitioned into K equal angular wedges. To quantify the marker distribution, we defined a polarity quantity vector as:

$$Z_{m}=\sum_{i=0}^{K-1}w_{i}e^{\text{i}m\theta_{i}}.$$

m: Number of poles (m=1 represents uni-polar, m=2 corresponds to bipolar organization)

$w_{i}:$ Mean intensity of wedge i

$\theta_{i}:$ Angular position around the cell

$e^{\text{i}m\theta_{i}}:$ Rotates each wedge’s intensity by $m\theta_{i}$ angle in the circular plane.

Therefore, $w_{i}e^{\text{i}m\theta_{i}}$ weighted direction for every wedge $w_{i}$ at an angle $m\theta_{i}$. Summing across all wedges, therefore, combines the full angular intensity pattern of the cell into a single polarity quantity $Z_{m}$.

To separate the strength of polarity from its direction, we defined the polarity magnitude $V_{m}$ and polarity angle $T_{m}$ as

$$V_{m}=\left|Z_{m}\right|\Delta \theta$$

$$T_{m}=\frac{\text{arg}\left(Z_{m}\right)}{m}$$

$\left|Z_{m}\right|$: Polarity Magnitude of the pole m

Δθ: Angle, normalize for the wedge size

$\text{arg}\left(Z_{m}\right)$: Angle of the polarity quantity vector $Z_{m}$

Multiplying Δθ by making it comparable across different values of total wedge number K.

#### Unipolar score:

To calculate the unipolar score m=1. This means each wedge contributes to the direction of its own antolar position $\theta_{i}$, weighted by its intensity $w_{i}$. As a result, $Z_{1}$ points toward the brighter side of the cell. Accordingly, $V_{1}$ measures the strength of the unipolar score, and $T_{1}$ gives its direction.

#### Bipolar score:

To calculate the bipolar score m=2, each wedge is evaluated at twice its angular position ($2\theta_{i}$) rather than at its original angle ($\theta_{i}$). This makes signals from opposite sides of the cell point in the same direction, allowing bipolar patterns to add constructively rather than cancel. For example, wedges at 0° and 180° both map to 0° after angle doubling, so signals from two opposite bright regions reinforce rather than cancel. Thus, $V_{2}$ measures the strength of bipolar organization, and $T_{2}$ defines the bipolar axis. By contrast, a purely bipolar pattern gives little or no m=1 signal, as vectors on opposing cell sides can cancel each other out.

#### Hemisphere Polarity Score:

As many cells are organized into two halves, CellPolariS can calculate the hemisphere polarity score to compare and measure the marker distribution on one side vs. the opposite side of the cell. This is particularly useful for live cell imaging of, for instance, migrating cells. For this, CellPolariS defines an axis ϕ that separates the two hemispheres using either a fixed user-defined angle, or an angle derived from the reference channel (see section: Correcting for cell motility during time-lapse imaging). CellPolariS then defines the two hemispheres 180° sectors centered at $\phi +90^{\circ }$ and $\phi +270^{\circ }$, corresponding to the left and right halves of the cell, respectively. For the angular intensity profile for every slice $w_{i}$ at angle $\theta_{i}$, we computed the left and right hemisphere amplitudes as

$$R_{L}=\left|\sum_{i}w_{i}\;H_{L}\left(\theta_{i};\phi \right)e^{\text{i}\theta_{i}}\right|\Delta \theta ,\;R_{R}=\left|\sum_{i}w_{i}\;H_{R}\left(\theta_{i};\phi \right)e^{\text{i}\theta_{i}}\right|\Delta \theta ,$$

where $H_{L}$ and $H_{R}$ are binary window functions selecting wedges belonging to the left and right hemispheres, respectively, $e^{\text{i}\theta_{i}}$ rotate the slice into a whole cell 360° and Δθ normalize for the wedge size. These quantities, therefore, measure the strength of signal enrichment within the two opposing halves of the circularized cell.

To place these values on an interpretable scale, we also expressed hemisphere amplitudes relative to a dynamic synthetic reference. Specifically, for each cell, we generated a synthetic half-cell reference with one bright hemisphere and one dim hemisphere and processed this reference using the same baseline correction applied to that cell. We then defined the reference amplitude, $R_{\text{ref}}$, as the larger of the two hemisphere amplitudes obtained from this baseline-adjusted synthetic reference. We calculated Percentage - normalized hemisphere scores as

$$R_{L}^{\left(\%\right)}=100\times \frac{R_{L}}{R_{\text{ref}}},R_{R}^{\left(\%\right)}=100\times \frac{R_{R}}{R_{\text{ref}}}.$$

Thus, $R_{L}$ and $R_{R}$ report the hemisphere amplitudes, whereas $R_{L}^{\left(\%\right)}$ and $R_{R}^{\left(\%\right)}$ report the corresponding amplitudes relative to the maximum hemisphere signal from a half-cell pattern under the same per-cell intensity correction.

### Correcting for cell motility during time-lapse imaging

To correct rotational cell motility during time-lapse imaging, CellPolariS defines a reference angle (direction) based on the center of mass of the user-selected reference channel at the first time point (e.g., a nuclear or cytoplasmic marker). For each subsequent time point, CellPolariS determines if the reference channel center of mass changes direction to calculate, correct, and apply the rotational offset compared to time point 1 to all imaging channels.

### Correcting for intensity changes in time-lapse imaging

To reduce time-dependent variation in background signal intensity, CellPolariS corrects background fluctuations by estimating and subtracting for each cell and channel the per-frame baseline intensity. The baseline was defined as

$$B=2M-P_{1}$$

where M is the modal intensity (the most frequent pixel intensity value in the frame) and $P_{1}$ is the 1st percentile of the intensity distribution. In practice, this estimates the background level by taking the dominant low-intensity peak of the image and correcting it using the lower tail of the distribution. The resulting baseline B was subtracted from the polarity vector w, and any negative values were clipped to zero.

### Statistical analysis

No statistical methods were used to predetermine sample size. The experiments were not randomized, and unless otherwise stated, the investigators were not blinded to allocation during experiments and outcome assessment. Unless otherwise stated, all experiments were repeated as independent replicates ≥3 times. All statistical tests used were two-sided. Unless stated differently, data were analyzed using a two-tailed Mann-Whitney U test. Mean ± standard error of the mean (SEM) is displayed using GraphPad Prism 11 and Python 3.10.19. Box plot elements are defined as: center line, median; box limits, upper and lower quartiles; Tukey‘s 1.5× interquartile range; points, outlier. Significance levels were as follows: *P < 0.05; **P < 0.01; ***P < 0.001.

### Use of Large Language Models

Generative AI tools (OpenAI ChatGPT/GPT-4+ and Anthropic Claude Opus 4.5+) were used as coding assistants. All generated code was reviewed, tested, and validated by the authors, who take full responsibility for the scientific content, analyses, and conclusions presented in this work.

### Data exclusion criteria

#### Immunostaining:

To improve data quality and remove imaging artifacts, we excluded segmented cells and/or objects touching the image boundary, containing saturated pixels, with an area less than 50 pixels or greater than 10,000 pixels, or without a detectable DAPI signal.

#### Time-lapse data:

Cells for live-cell polarity dynamics quantification were chosen randomly. Cells touching the image edge, overlapped, or in contact with other cells were excluded to prevent segmentation errors.

##### Critical reagents.

A list for critical reagents, including antibodies, key chemicals, cell lines, experimental models, oligonucleotides, and software, is provided in Supplementary Table 2.

##### Materials availability.

All unique reagents generated in this study are available with a completed Materials Transfer Agreement.

## Extended Data

## Supplementary Material

1 Supplementary Fig. 1: Flow cytometric gating strategy for isolation of hematopoietic stem and progenitor cells.

**Supplementary information:** The online version contains supplementary material available at XXX

Supplementary Figures and Tables

Supplementary Table 1 - List of antibodies used in this study

Supplementary Table 2 - Configuration of Cell Sorter used in this study

Supplementary Table 3 - Microscope Hardware used in this study

Supplementary Files

This is a list of supplementary files associated with this preprint. Click to download.

- SupplementaryTable1Listofantibodiesusedinthisstudy.xlsx
- SupplementaryFig.1.pdf
- SupplementaryTable2ConfigurationofCellSorterusedinthisstudy.xlsx
- SupplementaryTable3MicroscopeHardwareusedinthisstudy.xlsx

## Data Availability

Source data for Figs. 1–5 and Extended Data Figs. 1–9 are provided in Source Data .csv or .xlsx files. All other data supporting the findings of this study are available from the corresponding author on reasonable request.

## Code availability

The source code, sample datasets, pre-built Windows installer, and documentation for CellPolariS v0.1 are archived at Zenodo under the MIT License (https://doi.org/10.5281/zenodo.20404095). The Zenodo record is private during peer review and is accessible to editors and reviewers through the following private review link: https://zenodo.org/records/20404095?preview=1&token=eyJhbGciOiJIUzUxMiJ9.eyJpZCI6ImIzMzI1ZjBjLWRkODItNGM1Ni04ZDk1LTVmMGEyNGViZGFhNCIsImRhdGEiOnt9LCJyYW5kb20iOiJkMzBjNzg4YjZlMDQ5ZGNkMDdjYWY1MDIzZjIyMzBhMCJ9.e1DIeXqE7Y6NX8xp1opTOQIcs0lTBS7alI8HAGPw5NE_eNyj6Tcz73KEEzuDKPXDHG2rUJdVAKKDCqPb4pXozQ

The CellPolariS GitHub repository will be made publicly available upon publication.
