# Supplementary material for: Scalable quantification of dynamic subcellular spatial organization in single cells across tissues
Source: Res Sq. 2026 Jul 2:rs.3.rs-9916651. Preprint. [Version 1] doi: 10.21203/rs.3.rs-9916651/v1 (PMC13345535; doi:10.21203/rs.3.rs-9916651/v1)
Supplement: 1 — Supplementary Fig. 1: Flow cytometric gating strategy for isolation of hematopoietic stem and progenitor cells. [file NIHPPRS9916651V1-supplement-1.pdf]

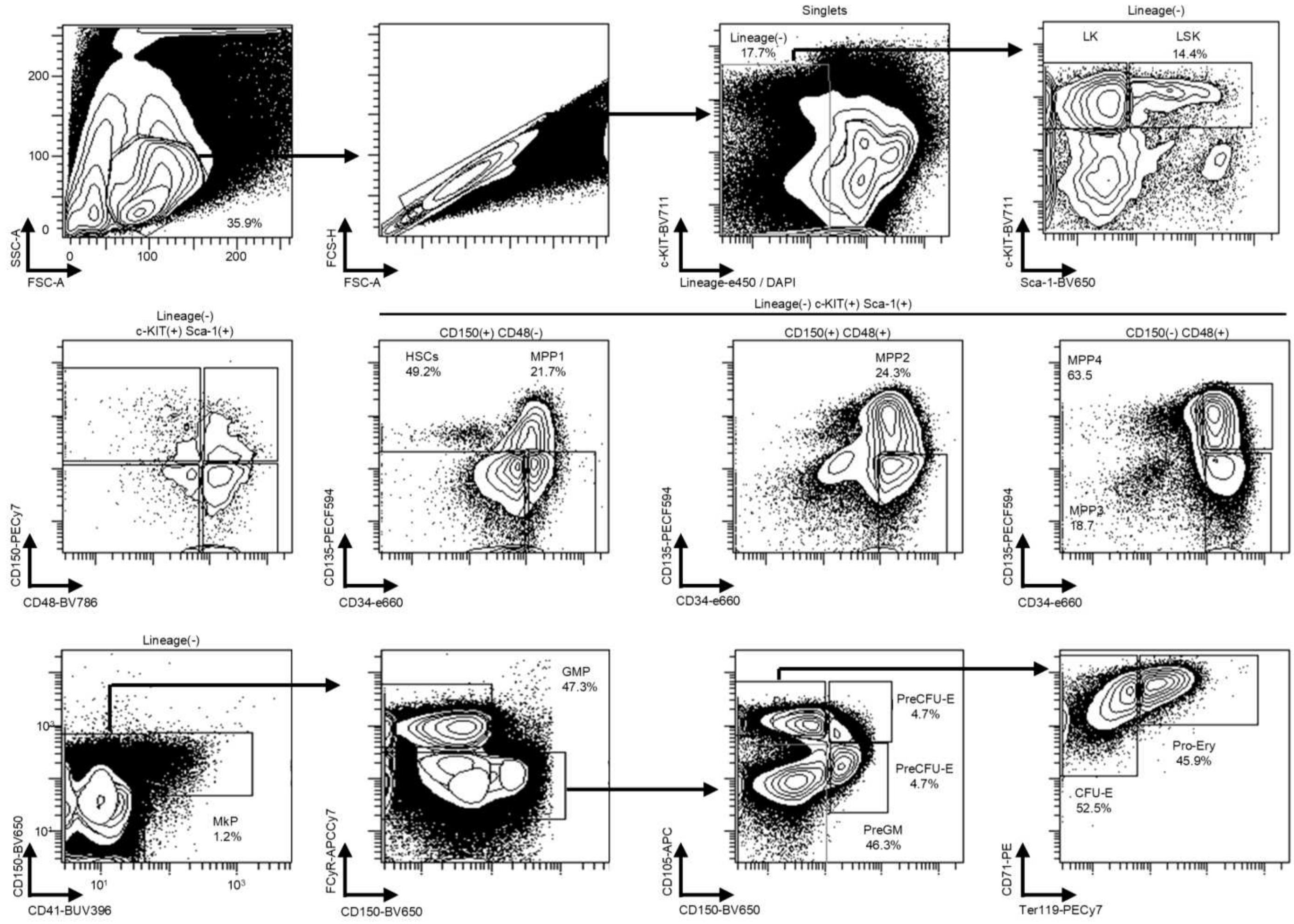

Supplementary Fig. 1: Flow cytometric gating strategy for isolation of hematopoietic stem and progenitor cells.

Flow cytometric sorting scheme used to isolate HSC and MPP1-5. (b) Flow cytometric gating strategy for isolation of progenitor cells. MkP – Megakaryocyte progenitor; GMP - Granulocyte-Monocyte Progenitor; PreCFUE – pre-Colony forming unit erythroid lineage; PreGm – pre-Granulocyte-Monocyte (myeloid) lineage; PreMegE – pre-Megakaryocyte- Erythrocyte lineage; ProEry. – Erythrocyte; CFU-E – Colony-forming unit erythroid lineage.
